# Salt stress memory in tall fescue: Interaction of different stress stages, pollination system and genetic diversity

**DOI:** 10.1371/journal.pone.0310061

**Published:** 2024-09-12

**Authors:** Maryam Safari, Mohammad Mahdi Majidi

**Affiliations:** Department of Agronomy and Plant Breeding, College of Agriculture, Isfahan University of Technology, Isfahan, Iran; Nuclear Science and Technology Research Institute, ISLAMIC REPUBLIC OF IRAN

## Abstract

**Introduction:**

The effects of salinity memory and its interaction with genetic diversity for drought tolerance and pollination system in terms of morphological, physiological, root characteristics and spectral reflectance indices (SRIs) in tall fescue is still unknown.

**Methods:**

Four tall fescue genotypes (two drought-sensitive and two drought-tolerant) were manually controlled to produce four selfed (S_1_) and four open-pollinated (OP) progeny genotypes (finally eight progeny genotypes). Then all genotypes were assessed for two years in greenhouse under five salinity treatments including control treatment (C), twice salinity stress treatment (primary mild salinity stress in two different stages and secondary at the end stage) (S_1t1_S_2_ and S_1t2_S_2_), once severe salinity stress treatment (secondary only, S_2_), and foliar spray of salicylic acid (SA) simultaneously with secondary salinity stress (H_2_S_2_).

**Results:**

Results indicated that obligate selfing (S_1_) caused to inbreeding depression in RWC, plant growth, catalase activity, root length and the ratio of root/shoot (R/S). Once salinity stress treatment (S_2_) led to depression in most measured traits, while pre-exposure to salinity (salinity memory) (S_1t1_S_2_ and S_1t2_S_2_) improved photosynthetic pigments, proline, antioxidant enzymes and R/S.

**Conclusion:**

Salinity memory was more pronounced in drought-sensitive genotypes, while it was more evident in OP than S_1_ population. Foliar spray of salicylic acid (SA) was almost equally effective in reducing the effects of salinity stress in both populations. The efficacy of application was more pronounced in tolerant genotypes compared to sensitive ones. The possibility of modeling correlated spectral reflectance indices (SRIs) for prediction of different morphological, physiological and root characteristics will be discussed.

## Introduction

Tall fescue (*Festuca arundinacea*) is an important grass species known for its ability to adapt to a variety of abiotic stresses, including salt, drought, and heat. This species is very interesting because it is able to withstand salt and drought stress, which limits plant growth and yields worldwide [1–3]. Recent studies have further explored the adaptive mechanisms of tall fescue to salt stress, highlighting its physiological and molecular responses [4]. Despite extensive research on salinity in plants, the specific mechanisms and factors affecting salt stress memory in tall fescue are poorly understood [2]. Addressing these knowledge gaps is important for developing resistant plant species that can withstand harsh environmental conditions [5]. Although much research has been conducted on the effect of salt stress on different plant species, there are still significant gaps, especially regarding the combined effects of different stages of stress and the effect of genetic background on salt stress memory in tall fescue.

Like the majority of the grass species, the presence of self-incompatibility, leading to cross-pollination, typically directs breeding efforts towards the creation of superior synthetic cultivars and the enhancement of diverse populations [6]. Self-pollination in outcrossing species results in fixing some alleles which may cause inbreeding depression [7]. Inbreeding usually refers to the intermating of closely related individuals, while "inbreeding depression" (ID) denotes the decrease in relative fitness observed in progenies compared to those resulting from outcrossing [8]. The magnitude of ID is depending on the genetic background of the species or population and the interaction of genotype by environment [9, 10]. Nevertheless, selfing can facilitate development of pure lines, a crucial aspect in constructing specialized populations for genetic studies. Also, selfing sometimes improves an attribute by gathering additive homozygous alleles together in progeny. Considering the importance of tall fescue as a hexaploidy and outcrossing grass species widely used for turf and forage purposes, little information is available about the fitness under selfing and cross-pollination and its interaction with stress memory and abiotic stresses.

These toxic effects and osmotic pressure caused by salinity stress significantly reduce the yield of crops cultivated in saline soils [11, 12]. Roots rely on shoots for carbohydrates, while shoots depend on roots for nutrients and water. Consequently, environmental ecological stresses may lead to an increase in the relative biomass of roots compared to shoots [13]. This shift, known as an increase in the Ratio of Root-to-Shoot (RSR), is a common response to salinity stress. Under salt stress, an augmented root biomass can facilitate the retention of toxic ions in root cellular tissues, preventing their translocation to other organs such as leaves. This mechanism contributes to the characteristic resistance of plants to salinity. However, it’s important to note that low water quality can impede plant maturation, leaf enlargement, and alter the link between the root and aerial parts [14]. In contrast to the well-studied changes in root system development under water stress, the understanding of how salinity influences the growth and mass of tall fescue root systems is not yet comprehensive [15, 16].

When plants encounter environmental stresses, their response is intricate and often involves significant alterations in both physiological processes and gene expression patterns [17]. Pre-exposure to environmental stress may produce appropriate reactions and alarm signal (such as physiology and gene expression) memorized by plants [18]. Most of these stress-induced changes are reset to the basal level once the stress is relieved, but some may be stable and carried forward as a form of “stress memory” [19, 20]. Previous research indicates that stress memory induced by pre-exposure to abiotic stresses, such as salt, cold, and hormonal treatments, can enhance the plant’s immune system and enables plants to cope more effectively with the subsequent stress events [18–20]. Ding *et al*. [18] reported that pre-exposure to drought could induce transcriptional changes in trainable genes in Arabidopsis. Sani *et al*. [21] expressed that in the primed plant, the level of methylation increased and as a result, the promoter cannot transcribe the gene related to the production of proline limiting enzyme. As a result, this gene will be turned off and proline will be produced. Therefore, a plant that has already experienced once the salinity stress can appear more and faster salt tolerance than a plant that has experienced it for the first time. On the other hand, some reports indicated that the application of plant growth regulators such as salicylic acid (SA) could improve stress tolerance in plants [22, 23]. In plants, salicylic acid (SA) is a signaling molecule synthesized endogenously, and it plays a pivotal role in various aspects of plant physiology, including growth, development, flowering, ripening, defense mechanisms, and responses to abiotic stresses [24, 25]. Applying certain growth regulators externally, such as epibrassinolide, salicylic acid, and glycinebetaine has been found to improve the salt tolerance of *Puccinellia distans* (weeping alkaligrass), *Lolium arundinacea* (tall fescue), and *Lolium perenne* (perennial ryegrass) [26–28].

To evaluate the reaction of plants to abiotic stresses, sampling techniques are mostly considered destructive, cost and time-inefficient and often do not provide real-time updates on the physiological status of plants. Moreover, their application on a large scale is often impractical [29]. A viable alternative to address these limitations is the utilization of remote sensing techniques coupled with visible (VIS)/near-infrared (NIR) spectroscopy. Remote sensing, with its ability to detect biochemical and physiological changes, allows for the indirect estimation of salt stress parameters in a fast manner compared to traditional methods [30, 31]. Salinity stress not only induces significant changes in plant water status but also negatively affects photosynthetic pigments and internal leaf structure. For instance, Poss *et al*. [32] demonstrated that the water index (WI = (R_900_/R_970_)), which incorporates a water absorption band from the NIR region (970 nm), reflects the response of *Agropyron elongatum* (wheatgrass) growth to salt stress. A meaningful correlation is observed between spectral properties and key plant metrics, such as biomass and leaf area index, in seashore paspalum (*Paspalum vaginatum*) [33] when cultivated in saline soils. Little information, however, is available in relation to the ability of spectral reflectance techniques to estimate physiological traits, antioxidant enzyme activities, root system and general performance of tall fescue.

The current study addressed important knowledge gaps regarding the effects of salt stress memory in different reproduce systems and genetic backgrounds in tall fescue. Although previous studies have mainly focused on the effects of salinity on plant characteristics and the independent effects of pollination systems or salicylic acid, our study provides the first joint analysis of these factors under different salinity conditions. This study explored the complex relationship between pollination systems (selfed vs. open-pollination), timing of stress memory application, salicylic acid treatment, and genetic variation in response to severe salinity stress, and examined how these factors influence morphological, physiological, root traits, and spectral reflectance indices. This comprehensive analysis is expected to provide new insights into improving salt stress management and address current knowledge gaps in the field.

## Materials and methods

### Plant materials and treatments

**Fig 1:**
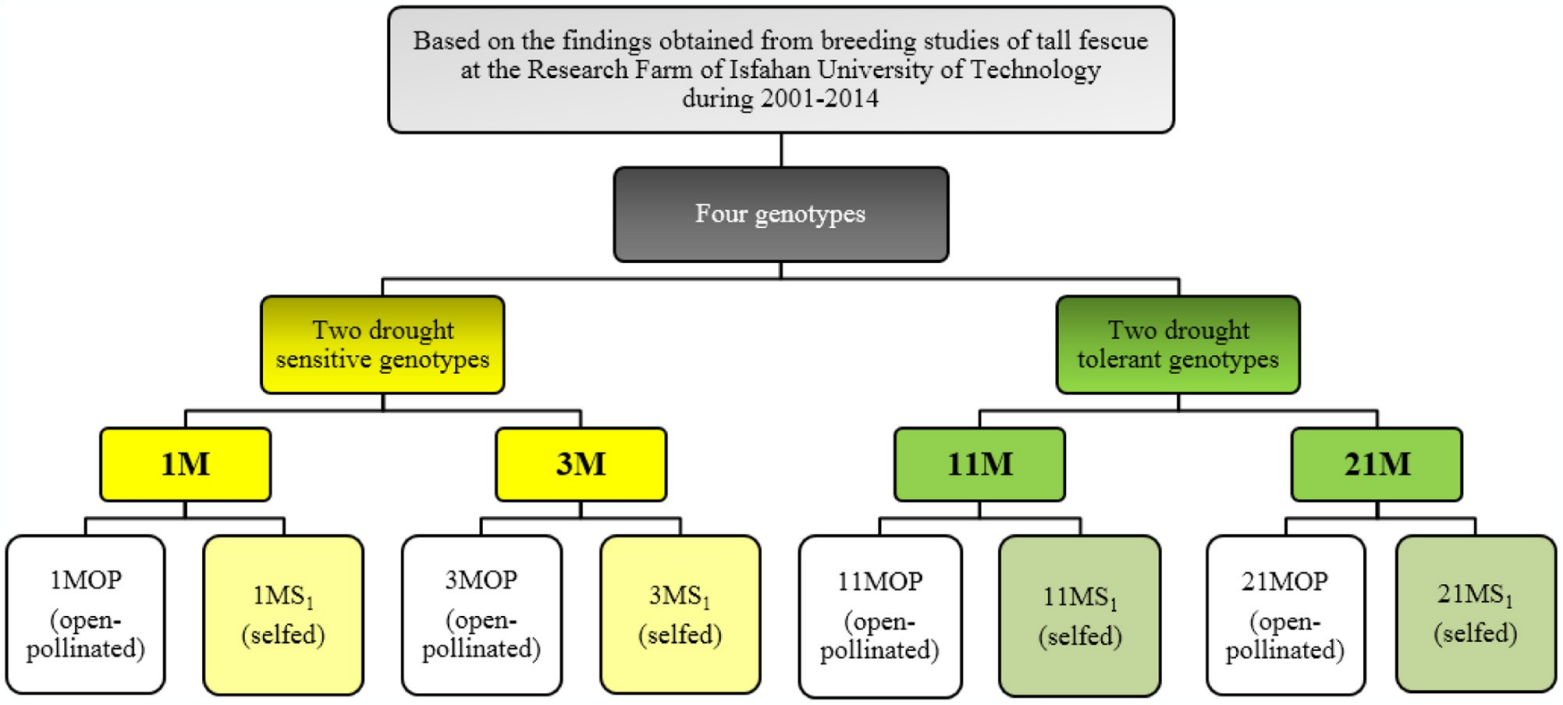
Information of tall fescue genotypes used in the study of salt tolerance.

The grass project was started in 2005 at Isfahan University of Technology. Over the years, 24 tall fescue genotypes have been collected within a large germplasm from numerous locations of Iran and a few other countries. The genotypes were selected based on morphological and drought tolerance indices in field as well as for root and physiological characteristics in pot experiments [34–37]. Finally, two drought tolerance (11M and 21M) and two susceptible genotypes (1M and 3M) were selected (Fig 1) and established in field.

In April 2020, half of the panicles of each genotype (parental plant) were isolated to ensure obligatory selfing, while the other half was left uncovered to allow open-pollination. By the end of the summer, the seeds from the selfed and open-pollinated panicles were harvested separately. Consequently, two populations were created, comprising 4 selfed progeny (S_1_) and 4 open-pollinated progeny (OP) (half-sib families), serving as the plant materials for the present study.

**Fig 2:**
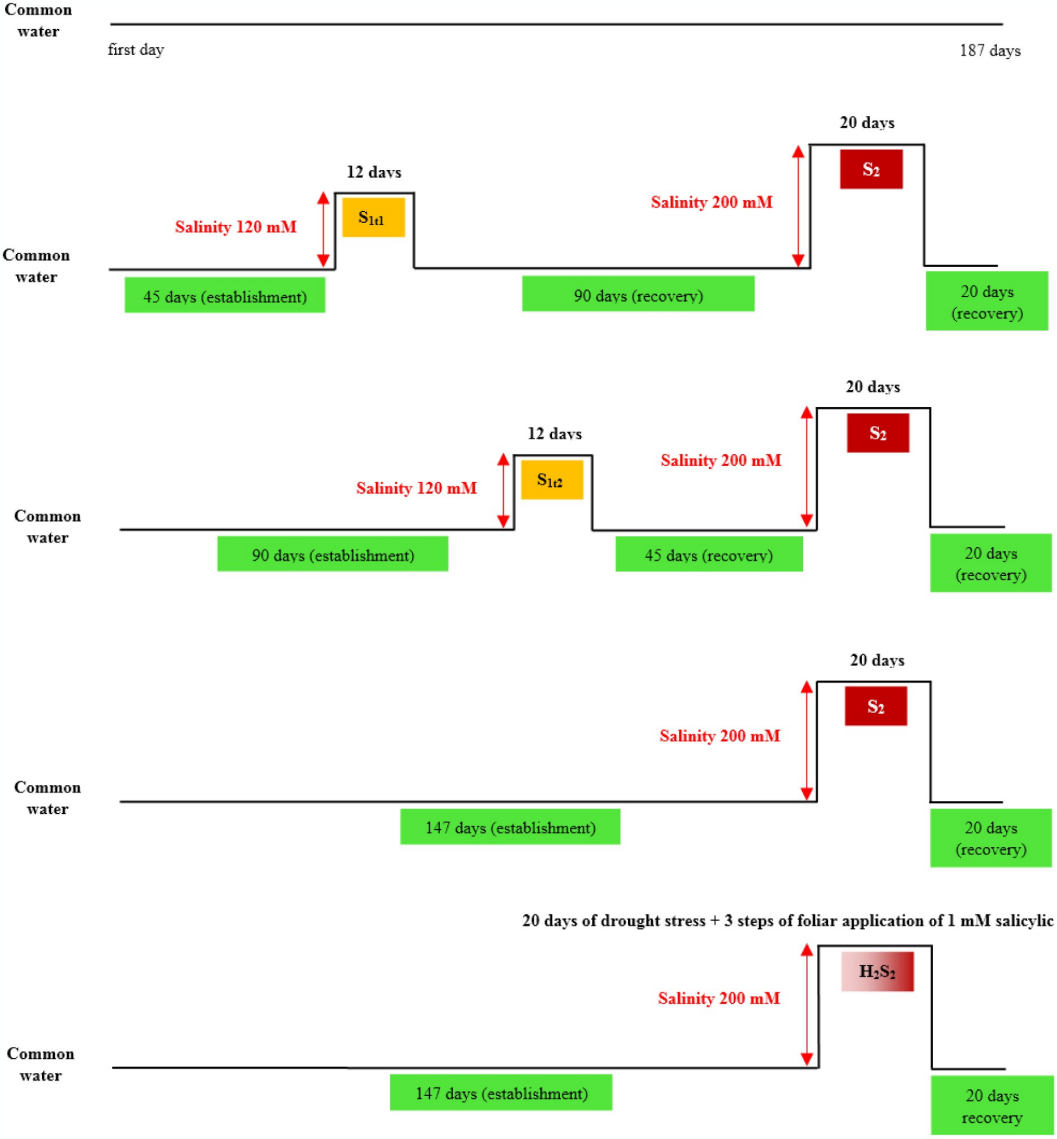
Steps of applying five salinity treatments (C, S_1t1_S_2_, S_1t2_S_2_, S_2_ and H_2_S_2_) of tall fescue genotypes in this study.

The seeds of S_1_ and OP populations were planted in pots in the research greenhouse of the Isfahan University of Technology. The plants were maintained in the greenhouse with 25/18°C (day/night), 14-h photoperiod, 75 ± 5% relative humidity, and an average photosynthetically active radiation of 400 μmol m^–2^ s^–1^. The experiment was conducted in autumn 2020 and repeated in 2021 according to a factorial arrangement based on a CRD with two replications. The first, second and third factors were genotypes (8 genotypes), pollination systems (2 levels), and salinity treatments (5 levels as follow), respectively. A mixture of silt loam soil and coarse sand (1:2 v/v) were used to fill the pots (35⨯18 cm). The weight of soil used in each pot was 7500 ± 50 g (pH = 7.4, BD = 1.32 g/cm^3^, and organic matter = 1.68%). After the complete establishment of the seedlings (about 45 days) salinity treatments (Fig 2) were applied as follows (salinity stress was applied gradually three times a week):

1. Control treatment **(C)**: Irrigation was conducted without limitation (normal condition) with common water.
2. Twice salinity stress treatment (primary in the first time and secondary at the end stage) **(S**_**1t1**_**S**_**2**_**)**: Primary mild salinity stress (120 mM NaCl saline) was supplied 45 days after establishment for 12 days (**S**_**1t1**_). Then plant allowed to recover for 90 days by common water and after that secondary stress (severe) applied with 200 mM NaCl saline for 20 days.
3. Twice salinity stress treatment (primary in the second time and secondary at the end stage) **(S**_**1t2**_**S**_**2**_**)**: Primary mild salinity stress (120 mM NaCl saline) was supplied 90 days after establishment for 12 days (**S**_**1t2**_). Then plant allowed to recover for 45 days by common water and after that secondary stress (severe) applied with 200 mM NaCl saline for 20 days.
4. Once salinity stress treatment (secondary only) **(S**_**2**_**)**: Only one severe salinity stress applied 147 days after establishment for 20 days with 200 mM NaCl saline.
5. Foliar spray of **SA** simultaneously with secondary salinity stress **(H**_**2**_**S**_**2**_**)**: This treatment was same as S_2_ with the difference that foliar salicylic acid (1 mM) was sprayed on the leaves during the stress (three times every three days) (Fig 2).

Irrigation of all pots was done depending on the needs of the plant and without considering the type of salinity treatment until the complete establishment (about 45 days). During this period, the seedlings were cut three times at two-week intervals to strengthen. Then, primary salinity stresses (S_1t1_ and S_1t2_) were implemented as mild salinity stress at two different stages as explained above (S1 Fig). Experienced plants with primary salinity stress, after a rest period (recovery), were treated with secondary salinity stress (severe stress) (S_1t1_S_2_ and S_1t2_S_2_). Once salinity stress treatment (S_2_) was also performed at this stage. Control treatment (C) was subjected to no stress until the end of the experiment. H_2_S_2_ treatment was implemented with secondary salinity stress and simultaneously salicylic acid (1 mM) was sprayed during stress. After the completion of mild salinity stress, some of the physiological traits (RWC, total chlorophyll, and proline) were measured. Several traits (including morphological (crown diameter, plant height, and forage yield) and physiological (RWC, photosynthetic pigments, proline, and antioxidant enzymes)) and spectroscopy were recorded for all treatments immediately after finishing severe stress. Measurement of morphological (plant height and forage yield) and root characteristics were performed at the end of the experiment (S1 Fig).

### Justification for the choice of factors and levels

The selection of factors and their levels in this study was carefully considered based on previous studies and the specific objectives of our research:

Genotypes (sensitive vs. tolerant):

Drought-sensitive genotypes: These genotypes were selected to understand how drought-sensitive plants respond to salinity stress, salinity memory and SA treatment. This helps determine possible improvements in tolerance.

Drought-tolerant genotypes: These genotypes was selected to see how natural plants benefit from salinity stress, stress memory and SA treatment, providing a basis for comparison and identification of specific genetic responses.

Pollination systems (selfed (S_1_) vs. open-pollinated (OP)):

The choice of selfed (S_1_) and open-pollinated (OP) systems was made to study the effects of genetic variation on salinity stress, salt stress memory, application of SA. Previous research has shown that mating system and genetic variations can significantly influence stress responses.

Salinity stress treatments (C, S_1t1_S_2_, S_1t2_S_2_, S_2_, H_2_S_2_):

C (control): Served as a control to compare the effects of severe salinity stress.

S_1t1_S_2_ (twice salinity stress treatment (primary in the first time and secondary at the end stage)): This treatment allowed us to evaluate the effect of early-stage mild salinity stress on the plant’s ability to withstand subsequent severe stress. It helps to understand if early exposure creates a stress memory that enhances tolerance.

S_1t2_S_2_ (twice salinity stress treatment (primary in the second time and secondary at the end stage)): This treatment tested whether the timing of mild stress exposure affected the plant’s stress memory and subsequent tolerance to severe stress, comparing early and later pre-exposure periods.

S_2_ (once severe salinity stress treatment): Serving as a treatment to understand the impact of severe salinity stress without any pre-exposure. This helps in comparing the effects of stress memory and SA application.

H_2_S_2_ (foliar spray of SA simultaneously with secondary salinity stress): Investigated the potential of SA to mitigate severe salinity stress by enhancing physiological and biochemical processes in the plant. This helps to understand the role of SA in stress tolerance.

These choices were designed to provide a comprehensive understanding of the interactions between different salt stress treatments, pollination systems, and genetic backgrounds. This method helps to identify the main factors and processes that lead to stress tolerance and memory of tall fescue.

### Measurements

Various morphological, physiological and Vis/NIR spectroscopy characteristics were measured in this study. Plant height (PH), crown diameter (plant width remaining after cut) (CRD) and wet and dry forage yield per plant (WFY and DFY) was recorded following the method of Pirnajmedin *et al*. [36].

Leaf water status was evaluated by estimating the Relative Water Content (RWC) following the method outlined by Ritchie *et al*. [38]. Additionally, spectrophotometry was utilized to measure total chlorophyll (Tchl), chlorophyll a (Chla), chlorophyll b (Chlb), and carotenoids (Car) [39]. Moreover, the determination of proline content (Pro) was carried out using the method outlined by Bates *et al*. [40]. To determine the protein content and evaluate the activity of the enzyme, a sample of 0.1 g of leaves was taken and subsequently preserved by freezing in liquid nitrogen. Catalase (CAT) activity was determined using the method described by Chang and Maehly (1995) [41]. Additionally, Ascorbate peroxidase (APX) and Peroxidase (POX) activity was mesured based on the methods of Nakano and Asada (1981), and Herzog and Fahimi (1973), respectively [42, 43]. Enzyme activities were quantified based on the weight of protein units, and the protein content was determined using bovine serum albumin as the standard, following the methodology outlined in the Bradford method [44].

To assess the root characteristic system, the roots were thoroughly washed to remove soil, and the root wet weight (RWW) was promptly measured. Root length (RL), root volume (RV(G)), root area (RA), and root cumulative length (RCL) were measured using a computer scanner and Gia Roots software [45]. Additionally, root volume (RV(A)) was determined using Archimedes’ law. Root dry weight (RDW) was obtained after drying the roots in an oven at 85°C for 48 hours. Finally, the root-to-shoot ratio (R/S) was calculated.

The Vis-NIR photo-diode array spectrometer (model: LR1 spectrometer, ASEQ Instrument, Vancouver, Canada) was employed to obtain the diffuse reflectance spectra of leaves of each genotype. Three leaves of each genotype and two parts of each leaf, finally six diffuse reflectance spectra for each genotype, were measured and the averaged spectrum of six spectra was used for statistical analysis. All measurements were performed within the confines of the greenhouse, with no alteration made to the tall fescue leaves. The relative reflectance spectrum was then utilized in the calculation of eighteen different indices, as listed in S1 Table for this study.

### Statistical analysis

The data underwent normality testing using the Q-Q plot test and were then subjected to analysis of variance (ANOVA) using SAS (2013) [46] to identify differences between years, genotypes, pollination systems, salinity treatments, and their interactions for each variable. Mean comparisons were conducted using the LSD test (*P* < 0.05) for various traits and spectral reflectance indices. Correlation coefficients between spectral reflectance indices and different traits were calculated using proc CORR in SAS [46]. Principal component analysis (PCA) was conducted on a correlation matrix [47] in order to decrease the numerous dimensions of the data space, utilizing Stat Graphics Centurion XVII [48]. The determination of the variables responsible for the majority of the variations in different traits was accomplished through stepwise multiple linear regression, employing SAS) [46].

## Results

### Analysis of variance and mean comparisons

Results from the analysis of variance for two years revealed that genotypes (G), pollination systems (P), and salinity treatments (T) had a significant effect (*P <* 0.05) on most of the measured traits (morphology, physiology and root) (S2–S4 Tables) and indices (not shown). The G×P, G×T, and P×T interaction effects and the triple interaction of G×P×T were also significant for most measured traits (S2–S4 Tables). The mean comparison of tall fescue genotypes, different pollination systems (selfed (S_1_) and open-pollinated (OP)), and five salinity treatments (C, S_1t1_S_2_, S_1t2_S_2_, S_2_, and H_2_S_2_) for different traits and indices are given in Tables 1–4.

**Table 1:** Mean comparison of tall fescue genotypes, two different pollination systems (selfed (S_1_) and open-pollinated (OP)) and five salinity treatments (C, S_1t1_S_2_, S_1t2_S_2_, S_2_ and H_2_S_2_) for morphological traits and relative water content.

	CRD (cm)	PH (cm)	RWC (%)	WFY (g/plant)	DFY (g/plant)	PHR (cm)	WFYR (g/plant)	DFYR (g/plant)
**Genotype**								
**1M**	3.57 ^d^	1.70 ^a^	50.01 ^c^	0.075 ^c^	0.055 ^d^	12.01 ^a^	0.141 ^d^	0.089 ^d^
**3M**	5.39 ^a^	11.92 ^b^	55.86 ^a^	0.097 ^b^	0.068 ^a^	9.31 ^c^	0.224 ^a^	0.143 ^a^
**11M**	4.80 ^b^	11.30 ^c^	56.01 ^a^	0.112 ^a^	0.066 ^b^	10.57 ^b^	0.218 ^b^	0.130 ^b^
**21M**	4.29 ^c^	13.55 ^a^	52.35 ^b^	0.112 ^a^	0.061 ^c^	11.77 ^a^	0.181 ^c^	0.111 ^c^
**Pollination system**								
**OP**	4.82 ^a^	13.15 ^a^	55.70 ^a^	0.078 ^b^	0.051 ^b^	11.97 ^a^	0.108 ^b^	0.061 ^b^
**S**_**1**_	4.21 ^b^	12.08 ^b^	51.42 ^b^	0.120 ^a^	0.074 ^a^	9.86 ^b^	0.274 ^a^	0.175 ^a^
**Salinity treatment**								
**C**	4.16 ^c^	12.34 ^c^	54.23 ^b^	0.098 ^b^	0.053 ^d^	12.12 ^a^	0.214 ^a^	0.125 ^b^
**S**_**1t1**_ **S**_**2**_	4.07 ^c^	14.25 ^a^	49.60 ^d^	0.112 ^a^	0.070 ^b^	9.00 ^d^	0.119 ^c^	0.078 ^d^
**S**_**1t2**_ **S**_**2**_	4.93 ^a^	13.06 ^b^	56.48 ^a^	0.099 ^b^	0.064 ^c^	11.34 ^b^	0.218 ^a^	0.133 ^a^
**S**_**2**_	4.71 ^b^	11.43 ^d^	55.05 ^b^	0.086 ^c^	0.051 ^e^	10.50 ^c^	0.203 ^b^	0.120 ^c^
**H**_**2**_ **S**_**2**_	4.68 ^b^	12.00 ^c^	52.45 ^c^	0.100 ^b^	0.076 ^a^	11.62 ^b^	0.199 ^b^	0.134 ^a^

Mean followed by the same letter is not significantly different according to LSD test (probability level of 5%).

CRD, crown diameter; PH, plant height; RWC, relative water content; WFY, wet forage yield; DFY, dry forage yield; PHR, plant height in recovery; WFYR, wet forage yield in recovery; DFYR, dry forage yield in recovery.

**Table 2:** Mean comparison of tall fescue genotypes, two different pollination systems (selfed (S_1_) and open-pollinated (OP)) and five salinity treatments (C, S_1t1_S_2_, S_1t2_S_2_, S_2_ and H_2_S_2_) for physiological traits.

	Chla (mg/g leaf)	Chlb (mg/g leaf)	Car (mg/g leaf)	Tchl (mg/g leaf)	Chl a/b	Tchl/Car	Pro (μmol/g leaf)	CAT (μmol min^-1^ mg^-1^ protein)	APX (μmol min^-1^ mg^-1^ protein)	POX (μmol min^-1^ mg^-1^ protein)
**Genotype**										
**1M**	0.502 ^a^	0.203 ^c^	0.215 ^a^	0.706 ^b^	2.43 ^b^	3.12 ^d^	2.17 ^a^	0.247 ^b^	0.054 ^a^	0.515 ^c^
**3M**	0.503 ^a^	0.229 ^a^	0.216 ^a^	0.733 ^a^	2.27 ^d^	3.43 ^a^	1.74 ^b^	0.310 ^a^	0.043 ^b^	0.288 ^a^
**11M**	0.494 ^a^	0.208 ^b^	0.216 ^a^	0.703 ^b^	2.35 ^c^	3.27 ^c^	1.65 ^c^	0.232 ^c^	0.027 ^c^	0.170 ^b^
**21M**	0.499 ^a^	0.199 ^c^	0.204 ^b^	0.699 ^b^	2.56 ^a^	3.34 ^b^	1.56 ^d^	0.237 ^bc^	0.028 ^c^	0.124 ^d^
**Pollination system**										
**OP**	0.464 ^b^	0.195 ^b^	0.199 ^b^	0.660 ^b^	2.33 ^b^	3.23 ^b^	1.61 ^b^	0.261 ^a^	0.035 ^b^	0.142 ^b^
**S**_**1**_	0.536 ^a^	0.225 ^a^	0.227 ^a^	0.761 ^a^	2.47 ^a^	3.35 ^a^	1.95 ^a^	0.252 ^b^	0.041 ^a^	0.225 ^a^
**Salinity treatment**										
**C**	0.687 ^a^	0.281 ^a^	0.252 ^a^	0.968 ^a^	2.44 ^c^	3.93 ^a^	1.65 ^c^	0.296 ^b^	0.031 ^c^	0.163 ^c^
**S**_**1t1**_ **S**_**2**_	0.572 ^b^	0.213 ^c^	0.204 ^c^	0.785 ^b^	2.79 ^a^	3.91 ^a^	1.89 ^a^	0.328 ^a^	0.042 ^b^	0.298 ^a^
**S**_**1t2**_ **S**_**2**_	0.542 ^c^	0.218 ^b^	0.233 ^b^	0.760 ^c^	2.50 ^b^	3.22 ^b^	1.91 ^a^	0.197 ^c^	0.044 ^a^	0.183 ^b^
**S**_**2**_	0.343 ^d^	0.147 ^e^	0.180 ^e^	0.490 ^e^	2.28 ^d^	2.65 ^d^	1.78 ^b^	0.144 ^d^	0.032 ^c^	0.141 ^d^
**H**_**2**_ **S**_**2**_	0.355 ^d^	0.192 ^d^	0.195 ^d^	0.547 ^d^	2.00 ^e^	2.75 ^c^	1.68 ^c^	0.318 ^a^	0.042 ^b^	0.132 ^e^

Mean followed by the same letter is not significantly different according to LSD test (probability level of 5%).

Chla, chlorophyll a content; Chlb, chlorophyll b content; Car, carotenoid content; Tchl, total chlorophyll; Chl a/b, ratio of Chla/Chlb; Tchl/Car, ratio of Tchl/Car; Pro, proline content; CAT, catalase activity; APX, ascorbate peroxidase activity; POX, peroxidase activity.

**Table 3:** Mean comparison of tall fescue genotypes, two different pollination systems (selfed (S_1_) and open-pollinated (OP)) and five salinity treatments (C, S_1t1_S_2_, S_1t2_S_2_, S_2_ and H_2_S_2_) for root traits.

	RL (cm)	RV(A) (cm^3^/plant)	RV(G) (cm^3^/plant)	RA (cm^2^/plant)	RCL (cm/plant)	RWW (g/plant)	RDW (g/plant)	R/S	R/SR
**Genotype**									
**1M**	18.25 ^d^	0.146 ^d^	0.214 ^c^	19.54 ^d^	174.70 ^d^	0.218 ^d^	0.083 ^d^	1.43 ^d^	0.670 ^d^
**3M**	26.90 ^a^	0.459 ^a^	0.448 ^a^	42.96 ^a^	390.40 ^a^	0.468 ^a^	0.141 ^a^	2.04 ^c^	0.858 ^c^
**11M**	24.45 ^b^	0.329 ^b^	0.453 ^a^	41.58 ^b^	374.34 ^b^	0.384 ^b^	0.119 ^b^	2.28 ^b^	1.34 ^b^
**21M**	20.72 ^c^	0.232 ^c^	0.237 ^b^	21.96 ^c^	200.26 ^c^	0.271 ^c^	0.112 ^c^	2.72 ^a^	2.03 ^a^
**Pollination system**									
**OP**	24.66 ^a^	0.162 ^b^	0.170 ^b^	16.60 ^b^	152.02 ^b^	0.212 ^b^	0.070 ^b^	1.83 ^b^	1.26 ^a^
**S**_**1**_	20.50 ^b^	0.420 ^a^	0.506 ^a^	46.42 ^a^	417.8 ^a^	0.459 ^a^	0.158 ^a^	2.41 ^a^	1.19 ^b^
**Salinity treatment**									
**C**	21.18 ^d^	0.195 ^d^	0.313 ^c^	28.75 ^bc^	259.52 ^b^	0.274 ^d^	0.083 ^e^	2.14 ^b^	0.537 ^e^
**S**_**1t1**_ **S**_**2**_	19.25 ^e^	0.318 ^b^	0.362 ^b^	35.06 ^a^	314.06 ^a^	0.350 ^b^	0.118 ^c^	1.76 ^c^	1.34 ^b^
**S**_**1t2**_ **S**_**2**_	24.37 ^b^	0.259 ^c^	0.298 ^d^	27.95 ^c^	259.99 ^b^	0.310 ^c^	0.102 ^d^	2.78 ^a^	2.59 ^a^
**S**_**2**_	22.78 ^c^	0.338 ^a^	0.396 ^a^	36.12 ^a^	322.23 ^a^	0.453 ^a^	0.140 ^a^	2.15 ^b^	0.786 ^d^
**H**_**2**_ **S**_**2**_	25.31 ^a^	0.347 ^a^	0.322 ^c^	29.66 ^b^	268.84 ^b^	0.289 ^d^	0.125 ^b^	1.77 ^c^	0.881 ^c^

Mean followed by the same letter is not significantly different according to LSD test (probability level of 5%).

RL, root length; RV(A), root volume (Archimedes); RV(G), root volume (Giaroot); RA, root area; RCL, root cumulative length; RWW, root wet weight; RDW, root dry weight; R/S, root to shoot ratio; R/SR, root to shoot ratio in recovery.

**Table 4:** Mean comparison of tall fescue genotypes, two different pollination systems (selfed (S_1_) and open-pollinated (OP)) and five salinity treatments (C, S_1t1_S_2_, S_1t2_S_2_, S_2_ and H_2_S_2_) for spectral reflectance indices.

	NDVI	SR	WI	RARSa	RARSb	PSSR	SIPI	NDRE	PSRI	CRI	ARI	GDVI	RGR
**Genotype**													
**1M**	1.00 ^b^	16.95 ^ab^	0.84 ^a^	0.17 ^c^	7.34 ^c^	9.54 ^b^	0.78 ^b^	0.22 ^b^	-0.072 ^b^	0.039 ^b^	-0.030 ^b^	44.43 ^b^	0.40 ^a^
**3M**	1.03 ^a^	12.18 ^c^	0.81 ^b^	0.21 ^a^	7.96 ^b^	8.52 ^c^	0.87 ^a^	0.24 ^a^	-0.071 ^b^	0.057 ^a^	-0.023 ^a^	42.45 ^d^	0.37 ^c^
**11M**	1.04 ^a^	18.24 ^a^	0.83 ^a^	0.19 ^b^	10.57 ^a^	10.77 ^a^	0.88 ^a^	0.24 ^a^	-0.074 ^b^	0.036 ^b^	-0.036 ^c^	43.13 ^c^	0.39 ^ab^
**21M**	0.98 ^b^	15.10 ^b^	0.84 ^a^	0.11 ^d^	10.27 ^a^	9.66 ^b^	0.86 ^a^	0.24 ^a^	-0.059 ^a^	0.037 ^b^	-0.028 ^b^	46.91 ^a^	0.38 ^b^
**Pollination system**													
**OP**	1.03 ^a^	15.81 ^a^	0.83 ^a^	0.22 ^a^	9.45 ^a^	9.10 ^b^	0.89 ^a^	0.24 ^a^	-0.058 ^a^	0.040 ^b^	-0.033 ^b^	43.22 ^a^	0.39 ^a^
**S**_**1**_	0.99 ^b^	15.42 ^a^	0.84 ^a^	0.12 ^b^	8.63 ^b^	10.15 ^a^	0.81 ^b^	0.23 ^b^	-0.080 ^b^	0.045 ^a^	-0.026 ^a^	45.24 ^b^	0.38 ^a^
**Salinity treatment**													
**C**	0.90 ^b^	21.71 ^a^	0.88 ^b^	0.059 ^c^	6.69 ^d^	7.29 ^c^	0.74 ^b^	0.20 ^c^	-0.097 ^d^	0.053 ^a^	-0.024 ^c^	44.38 ^c^	0.25 ^d^
**S**_**1t1**_ **S**_**2**_	0.81 ^c^	9.96 ^b^	1.01 ^a^	0.045 ^d^	6.37 ^d^	6.08 ^d^	0.67 ^c^	0.20 ^c^	-0.064 ^c^	0.028 ^d^	-0.011 ^a^	46.32 ^a^	0.47 ^c^
**S**_**1t2**_ **S**_**2**_	1.22 ^a^	10.19 ^b^	0.75 ^d^	0.311 ^b^	10.45 ^b^	15.81 ^a^	1.05 ^a^	0.26 ^b^	-0.054 ^b^	0.047 ^ab^	-0.049 ^e^	41.68 ^e^	0.50 ^b^
**S**_**2**_	0.92 ^b^	24.02 ^a^	0.77 ^c^	0.047 ^d^	8.66 ^c^	6.29 ^d^	0.70 ^bc^	0.21 ^c^	-0.090 ^d^	0.040 ^c^	-0.020 ^b^	45.40 ^b^	0.18 ^e^
**H**_**2**_ **S**_**2**_	1.21 ^a^	12.20 ^b^	0.76 ^c^	0.408 ^a^	13.02 ^a^	12.63 ^b^	1.08 ^a^	0.29 ^a^	-0.039 ^a^	0.043 ^bc^	-0.042 ^d^	43.38 ^d^	0.53 ^a^

Mean followed by the same letter is not significantly different according to LSD test (probability level of 5%).

NDVI, normalized difference vegetation index; SR, simple ratio; WI, water index; RARSa, ratio analysis of reflectance spectra; RARSb, ratio analysis of reflectance spectra; PSSR, pigment specific simple ratio; SIPI, structure intensive pigment index; NDRE, normalized difference red edge index; PSRI, plant senescence reflectance index; CRI, cartenoid reflectance index; ARI, anthocyanin reflectance index; GDVI, Green difference vegetation index; RGR, red/ green ratio.

#### How did different pollination systems (selfed (S_1_) and open-pollinated (OP)) affect traits

Mean comparison of pollination systems on measured traits showed that open-pollinated (OP) led to enhancement of crown diameter (CRD), plant height (PH), relative water content (RWC), plant height in recovery (PHR), catalase (CAT) activity, root length (RL) and ratio of root/shoot in recovery (R/SR) compared to selfed system (Tables 1–3). While selfing enhanced dry forage yield (DFY), chlorophyll a (Chla), chlorophyll b (Chlb), carotenoid content (Car), total chlorophyll (Tchl), ratio of Chl a/b (Chl a/b), ratio of the TChl/Car (TChl/Car), proline content (Pro), ascorbate peroxidase (APX), peroxidase (POX) activities and most measured root traits compared to open-pollinated (Tables 1–3). The emergence rate of selfed genotypes was lower than open-pollinated ones (S2 Fig), indicating evidence for inbreeding depression in the early stages of growth. However within S_1_ and OP populations, genotypes showed considerable variation. For example within S_1_ population, genotypes 1M and 11M had highest emergence rate, and genotypes 3M and 21M had the lowest value of this trait (S2 Fig).

The correlation coefficients among spectral reflectance indices were calculated and are presented in S5 Table. To reduce the number of indices for subsequent analyses, 5 indices, RNDVI, NWI, PRI, PSND, and GNDVI, were removed due to their high correlations with other indices. Results of the mean comparison of pollination systems for spectral reflectance indices indicated that the highest amount of normalized difference vegetation index (NDVI), ratio analysis of reflectance spectra (RARSa), ratio analysis of reflectance spectra (RARSb), structure intensive pigment index (SIPI), normalized difference red edge index (NDRE), plant senescence reflectance index (PSRI) and green difference vegetation index (GDVI) and the lowest amount of PSSR, CRI and ARI were observed in OP compared with S_1_ genotypes (Table 4).

#### How did severe salinity stress (S_2_) affect traits

The mean comparison of salinity treatments showed that severe final salinity stress (S_2_) significantly decreased PH, WFY, DFY, PHR, WFYR, DFYR, Chla, Chlb, Car, Tchl, Chl a/b, Tchl/Car and CAT activity (Tables 1 and 2). While Pro and all root traits were increased under S_2_ treatment (Tables 2 and 3). Results of the mean comparison of salinity treatments for indices represented that severe stress (S_2_) significantly reduced WI, RARSa, pigment specific simple ratio (PSSR), cartenoid reflectance index (CRI) and red/ green ratio (RGR) compared to control treatment (Table 4).

#### How did the timing of stress memory application and foliar spray of salicylic acid (SA) affect traits

**Fig 3:**
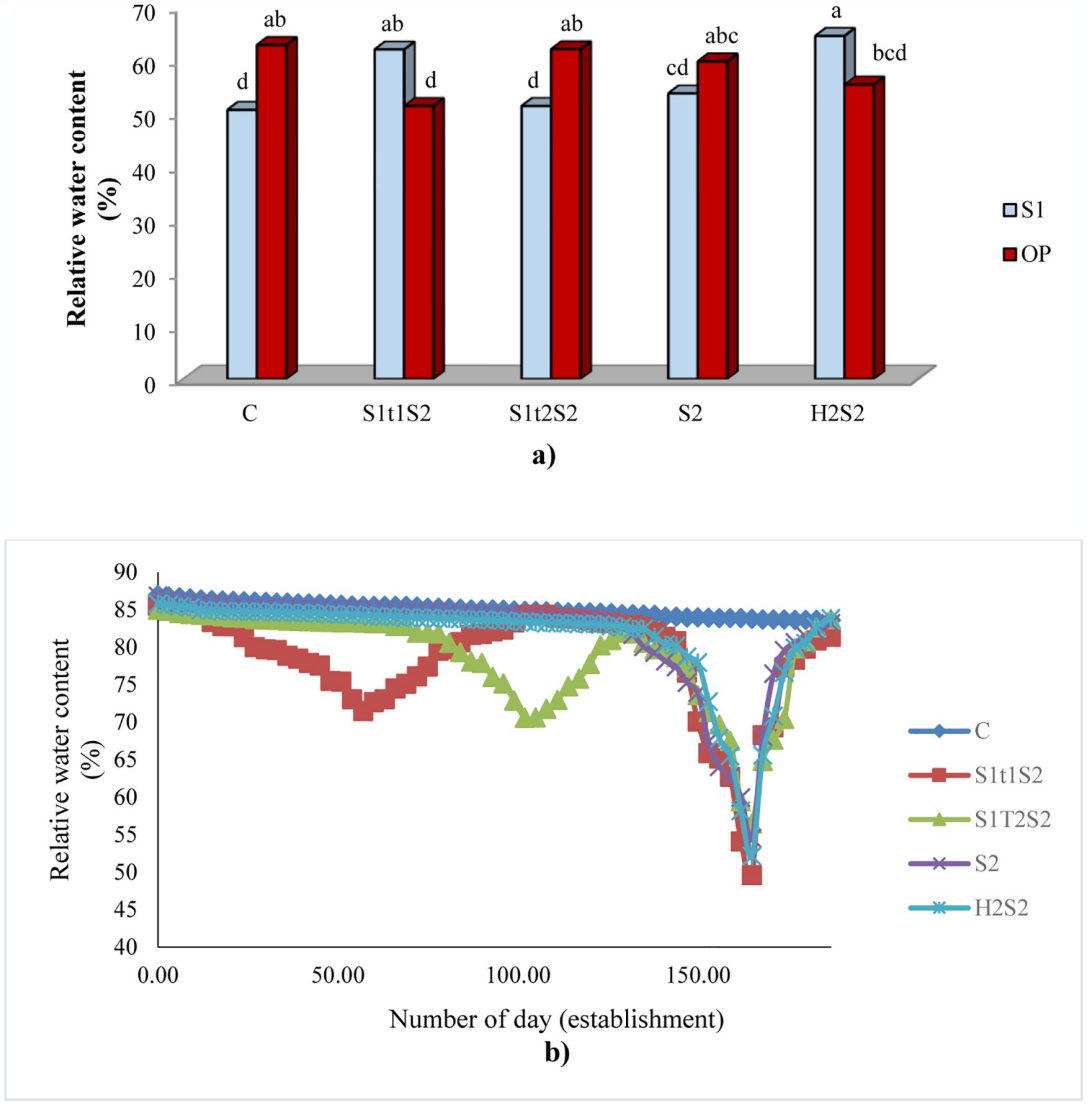
Mean comparison relative water content for the interactions of two different pollination systems (selfed (S_1_) and open-pollinated (OP)) and five salinity treatments (C, S_1t1_S_2_, S_1t2_S_2_, S_2_ and H_2_S_2_) during two years (a). Mean followed by the same letter is not significantly different according to LSD test (probability level of 5%). The trend of relative water content for five salinity treatments (C, S_1t1_S_2_, S_1t2_S_2_, S_2_ and H_2_S_2_) based on the number of day (establishment) during two years (b).

In this study, we applied stress memory in two different stages (45 and 90 days after establishment, S_1t1_S_2_ and S_1t2_S_2_, respectively) to see how the stage of applying affects this phenomenon. The results showed that similar trends were observed in both stages in terms of measured traits except for RWC, PHR, WFYR, DFYR, RL and R/S (Tables 1–3). Pre-exposure to salinity for causing salinity memory (S_1t1_S_2_ and S_1t2_S_2_) significantly increased PH, WFY, DFY and all physiological traits and R/SR compared to S_2_ treatment (Tables 1–3). Thus, it can be said that salinity memory was more evident for morphological and physiological traits. Results of the mean comparison for those physiological traits measured immediately after the first salinity stress condition (S_1_), at two different times, (S_1t1_ and S_1t2_) indicated that RWC and Tchl decreased and Pro enhanced compared with control treatment in both pollination systems (OP and S_1_) (S3a–S3f Fig). These results confirmed that the primary salinity stress was correctly applied and caused salinity memory in the secondary stress. For example, the S_1t1_S_2_ treatment in S_1_ increased RWC compared with the S_2_ treatment which indicated the existence of salinity memory (Fig 3a). Results of the mean comparison of for indices showed that under S_1t1_S_2_ treatment considerably enhanced WI, PSRI, ARI, GDVI and RGR compared to S_2_ treatment. Similarly, NDVI, RARSa, RARSb, PSSR, SIPI, NDRE, PSRI, CRI and RGR were increased under S_1t2_S_2_ treatment (Table 4).

Foliar spray of salicylic acid (SA) significantly improved most measured morphological and physiological traits, RL and R/SR compared to S_2_ treatment (Tables 1–3). Foliar application of SA at secondary salinity stress (H_2_S_2_) significantly enhanced RWC in tall fescue selfed genotypes compared to S_2_ treatment (Fig 3a). The trend of relative water content for five salinity treatments (C, S_1t1_S_2_, S_1t2_S_2_, S_2_ and H_2_S_2_) based on the number of day (establishment) during two years is shown in Fig 3b. At the end of the applied S_2_ stress, on the 165th day, C, S_1t2_S_2_, S_2_ and H_2_S_2_ treatments had the highest RWC respectively, and the S_1t1_S_2_ treatment had the lowest ones (Fig 3b). Results of the mean comparison of indices indicated exogenous application of SA lead to enhancement of NDVI, RARSa, RARSb, PSSR, SIPI, NDRE, PSRI and RGR under severe salinity stress compared with S_2_ treatment (Table 4).

### Interaction effects of different treatments

Mean comparison for interaction effect of salinity treatments and pollination systems for some morphological, physiological, root characteristics and the indices of spectral reflectance are presented in Tables 5 and 6.

**Table 5:** Mean comparison of interactions between five salinity treatments (C, S_1t1_S_2_, S_1t2_S_2_, S_2_ and H_2_S_2_) and two different pollination systems (selfed (S_1_) and open-pollinated (OP)) for some morphological, physiological and root traits.

**Morphological traits**												
**Treatment**	PH (cm)		RWC (%)		WFY (g/plant)		DFY (g/plant)		PHR (cm)		DFYR (g/plant)	
	**OP**	**S**_**1**_	**OP**	**S**_**1**_	**OP**	**S**_**1**_	**OP**	**S**_**1**_	**OP**	**S**_**1**_	**OP**	**S**_**1**_
**C**	14.75 ^a^	9.93 ^d^	60.45 ^a^	48.01 ^de^	0.123 ^cd^	0.072 ^e^	0.062 ^cd^	0.044 ^e^	15.06 ^a^	9.18 ^cde^	0.081 ^d^	0.169 ^b^
**S**_**1t1**_ **S**_**2**_	15.62 ^a^	12.87 ^bc^	46.61 ^e^	52.58 ^cd^	0.074 ^e^	0.150 ^a^	0.061 ^cd^	0.078 ^b^	8.37 ^e^	9.62 ^cde^	0.036 ^f^	0.120 ^c^
**S**_**1t2**_ **S**_**2**_	13.00 ^bc^	13.12 ^b^	62.14 ^a^	50.81 ^cde^	0.070 ^e^	0.129 ^bc^	0.043 ^e^	0.085 ^b^	11.81 ^b^	10.87 ^bc^	0.066 ^e^	0.201 ^a^
**S**_**2**_	9.87 ^d^	13.00 ^bc^	58.72 ^ab^	51.37 ^cde^	0.057 ^f^	0.115 ^d^	0.034 ^f^	0.068 ^c^	10.25 ^bcde^	10.75 ^bcd^	0.063 ^e^	0.178 ^b^
**H**_**2**_ **S**_**2**_	12.50 ^bc^	11.50 ^c^	50.59 ^cde^	54.31 ^bc^	0.066 ^ef^	0.134 ^b^	0.057 ^d^	0.095 ^a^	14.37 ^a^	8.87 ^de^	0.061 ^e^	0.207 ^a^
Mean	13.15 ^A^	12.08 ^B^	55.70 ^A^	51.42 ^B^	0.078 ^B^	0.120 ^A^	0.051 ^B^	0.074 ^A^	11.97 ^A^	9.86 ^B^	0.061 ^B^	0.175 ^A^
**Physiological traits**												
**Treatment**	Car (mg/g leaf)		Tchl (mg/g leaf)		Pro (μmol/g leaf)		CAT (μmol min^-1^ mg^-1^ protein)		APX (μmol min^-1^ mg^-1^ protein)		POX (μmol min^-1^ mg^-1^ protein)	
	**OP**	**S**_**1**_	**OP**	**S**_**1**_	**OP**	**S**_**1**_	**OP**	**S**_**1**_	**OP**	**S**_**1**_	**OP**	**S**_**1**_
**C**	0.261 ^a^	0.243 ^ab^	1.01 ^a^	0.925 ^b^	1.64 ^cde^	1.67 ^cde^	0.228 ^d^	0.364 ^b^	0.026 ^d^	0.035 ^c^	0.101 ^g^	0.224 ^bc^
**S**_**1t1**_ **S**_**2**_	0.172 ^e^	0.235 ^bc^	0.648 ^c^	0.921 ^b^	1.46 ^e^	2.31 ^a^	0.280 ^c^	0.376 ^b^	0.056 ^a^	0.028 ^d^	0.193 ^cd^	0.403 ^a^
**S**_**1t2**_ **S**_**2**_	0.211 ^cd^	0.254 ^ab^	0.666 ^c^	0.855 ^b^	1.84 ^bc^	1.97 ^b^	0.168 ^e^	0.225 ^d^	0.029 ^d^	0.059 ^a^	0.120 ^fg^	0.246 ^b^
**S**_**2**_	0.170 ^e^	0.190 ^de^	0.474 ^d^	0.506 ^d^	1.54 ^de^	2.01 ^b^	0.147 ^e^	0.141 ^e^	0.031 ^cd^	0.032 ^cd^	0.167 ^de^	0.115 ^fg^
**H**_**2**_ **S**_**2**_	0.180 ^e^	0.211 ^cd^	0.500 ^d^	0.594 ^c^	1.58 ^cde^	1.78 ^bcd^	0.481 ^a^	0.156 ^e^	0.035 ^c^	0.049 ^b^	0.129 ^fg^	0.136 ^ef^
Mean	0.199 ^B^	0.227 ^A^	0.660 ^B^	0.761 ^A^	1.61 ^B^	1.95 ^A^	0.261 ^A^	0.252 ^B^	0.035 ^B^	0.041 ^A^	0.142 ^B^	0.225 ^A^
**Root traits**												
**Treatment**	RV(G) (cm^3^/plant)		RA (cm^2^/plant)		RCL (cm/plant)		RDW (g/plant)		R/S		R/SR	
	**OP**	**S**_**1**_	**OP**	**S**_**1**_	**OP**	**S**_**1**_	**OP**	**S**_**1**_	**OP**	**S**_**1**_	**OP**	**S**_**1**_
**C**	0.237 ^e^	0.388 ^d^	21.93 ^e^	35.56 ^d^	200.4 ^e^	318.5 ^d^	0.060 ^f^	0.106 ^d^	1.75 ^d^	2.52 ^b^	0.657 ^de^	0.417 ^f^
**S**_**1t1**_ **S**_**2**_	0.176 ^f^	0.547 ^b^	20.05 ^e^	50.07 ^b^	179.7 ^ef^	448.4 ^b^	0.082 ^e^	0.15 ^bc^	2.02 ^c^	1.49 ^e^	1.94 ^b^	0.749 ^cde^
**S**_**1t2**_ **S**_**2**_	0.197 ^ef^	0.400 ^d^	37.11 ^d^	18.80 ^ef^	177.5 ^ef^	342.4 ^d^	0.060 ^f^	0.144 ^c^	1.65 ^de^	3.91 ^a^	1.87 ^b^	3.30 ^a^
**S**_**2**_	0.076 ^g^	0.715 ^a^	7.04 ^g^	65.21 ^a^	64.31 ^g^	580.1 ^a^	0.064 ^ef^	0.216 ^a^	2.50 ^b^	1.80 ^cd^	0.953 ^c^	0.619 ^ef^
**H**_**2**_ **S**_**2**_	0.164 ^f^	0.479 ^c^	15.19 ^f^	44.13 ^c^	138.1 ^f^	399.5 ^c^	0.081 ^e^	0.168 ^b^	1.23 ^f^	2.31 ^b^	0.898 ^c^	0.864 ^cd^
Mean	0.170 ^B^	0.506 ^A^	16.60 ^B^	46.42 ^A^	152.0 ^B^	417.8 ^A^	0.070 ^B^	0.158 ^A^	1.83 ^B^	2.41 ^A^	1.26 ^A^	1.19 ^B^

Mean followed by the same letter is not significantly different according to LSD test (probability level of 5%).

PH, plant height; RWC, relative water content; WFY, wet forage yield; DFY, dry forage yield; PHR, plant height in recovery; DFYR, dry forage yield in recovery; Car, carotenoid content; Tchl, total chlorophyll; Pro, proline content; CAT, catalase activity; APX, ascorbate peroxidase activity; POX, peroxidase activity; RV(A), root volume (Archimedes); RA, root area; RCL, root cumulative length; RDW, root dry weight; R/S, root to shoot ratio; R/SR, root to shoot ratio in recovery.

**Table 6:** Mean comparison of interactions between five salinity treatments (C, S_1t1_S_2_, S_1t2_S_2_, S_2_ and H_2_S_2_) and two different pollination systems (selfed (S_1_) and open-pollinated (OP)) for some spectral reflectance indices.

**Spectral reflectance indices**												
**Treatment**	NDVI		SR		WI		RARSa		RARSb		PSSR	
	**OP**	**S**_**1**_	**OP**	**S**_**1**_	**OP**	**S**_**1**_	**OP**	**S**_**1**_	**OP**	**S**_**1**_	**OP**	**S**_**1**_
**C**	0.89 ^d^	0.92 ^d^	24.65 ^ab^	18.78 ^cd^	0.904 ^b^	0.856 ^c^	0.043 ^f^	0.075 ^e^	7.12 ^d^	6.25 ^ef^	7.45 ^d^	7.14 ^de^
**S**_**1t1**_ **S**_**2**_	0.81 ^e^	0.80 ^e^	10.30 ^e^	9.62 ^e^	1.000 ^a^	1.022 ^a^	0.049 ^f^	0.041 ^f^	6.73 ^def^	6.00 ^f^	6.25 ^ef^	5.92 ^f^
**S**_**1t2**_ **S**_**2**_	1.24 ^a^	1.20 ^b^	9.26 ^e^	11.12 ^e^	0.739 ^f^	0.761 ^ef^	0.385 ^b^	0.238 ^c^	11.15 ^b^	9.75 ^c^	13.27 ^b^	18.35 ^a^
**S**_**2**_	0.93 ^d^	0.90 ^d^	26.55 ^a^	21.49 ^bc^	0.773 ^de^	0.774 ^de^	0.046 ^f^	0.048 ^f^	10.37 ^c^	6.95 ^de^	6.61 ^def^	5.98 ^f^
**H**_**2**_ **S**_**2**_	1.28 ^a^	1.13 ^c^	8.31 ^e^	16.09 ^d^	0.748 ^f^	0.783 ^d^	0.607 ^a^	0.210 ^d^	11.86 ^b^	14.18 ^a^	11.90 ^c^	13.36 ^b^
Mean	1.03 ^A^	0.99 ^B^	15.81 ^A^	15.42 ^A^	0.833 ^A^	0.839 ^A^	0.226 ^A^	0.122 ^B^	9.45 ^A^	8.63 ^B^	9.10 ^B^	10.15 ^A^
**Spectral reflectance indices**												
**Treatment**	SIPI		PSRI		CRI		ARI		GDVI		RGR	
	**OP**	**S**_**1**_	**OP**	**S**_**1**_	**OP**	**S**_**1**_	**OP**	**S**_**1**_	**OP**	**S**_**1**_	**OP**	**S**_**1**_
**C**	0.71 ^cd^	0.75 ^c^	-0.096 ^e^	-0.098 ^e^	0.046 ^b^	0.059 ^a^	-0.022 ^c^	-0.027 ^b^	45.50 ^b^	43.25 ^c^	0.230 ^e^	0.271 ^d^
**S**_**1t1**_ **S**_**2**_	0.67 ^d^	0.66 ^d^	-0.059 ^b^	-0.069 ^bc^	0.031 ^de^	0.024 ^e^	-0.010 ^a^	-0.013 ^ab^	45.60 ^b^	47.04 ^a^	0.481 ^c^	0.465 ^c^
**S**_**1t2**_ **S**_**2**_	1.13 ^a^	0.97 ^b^	-0.032 ^a^	-0.075 ^cd^	0.035 ^cd^	0.060 ^a^	-0.059 ^h^	-0.039 ^f^	42.02 ^d^	41.34 ^d^	0.484 ^c^	0.530 ^b^
**S**_**2**_	0.75 ^c^	0.66 ^d^	-0.083 ^d^	-0.098 ^e^	0.046 ^b^	0.035 ^cd^	-0.023 ^cd^	-0.017 ^b^	43.47 ^c^	47.32 ^a^	0.208 ^e^	0.166 ^f^
**H**_**2**_ **S**_**2**_	1.17 ^a^	0.98 ^b^	-0.020 ^a^	-0.058 ^b^	0.041 ^bc^	0.045 ^b^	-0.052 ^g^	-0.032 ^e^	39.52 ^e^	47.24 ^a^	0.588 ^a^	0.465 ^c^
Mean	0.89 ^A^	0.81 ^B^	-0.058 ^A^	-0.080 ^B^	0.040 ^B^	0.045 ^A^	-0.033 ^B^	-0.026 ^A^	43.22 ^A^	45.24 ^B^	0.390 ^A^	0.389 ^A^

Mean followed by the same letter is not significantly different according to LSD test (probability level of 5%).

NDVI, normalized difference vegetation index; SR, simple ratio; WI, water index; RARSa, ratio analysis of reflectance spectra; RARSb, ratio analysis of reflectance spectra; PSSR, pigment specific simple ratio; SIPI, structure intensive pigment index; PSRI, plant senescence reflectance index; CRI, cartenoid reflectance index; ARI, anthocyanin reflectance index; GDVI, Green difference vegetation index; RGR, red/ green ratio.

#### How did severe salinity stress affect different pollination systems in tall fescue

In open-pollinated genotypes, substantial reductions in PH, WFY, DFY, DFYR, Pro, RV(G), RA, RCL and RDW were observed under severe salinity stress compared to selfed genotypes (Table 5). As a result, it can be concluded that severe salinity stress had more negative effects on these genotypes. Meanwhile, selfed genotypes exhibited notable declines in most spectral reflectance indices (Table 6).

#### How did different pollination systems influence the effectiveness of stress memory and application of SA in tall fescue

In open-pollination, results showed significant increases in PH, WFY, DFY, Tchl, RV(G), RA, RCL and R/SR under memory treatments (S_1t1_S_2_ and S_1t2_S_2_) compared to non-memory treatment (S_2_) (Table 5). Meanwhile, CAT and APX activities under S_1t1_S_2_ treatment and Car and Pro under S_1t2_S_2_ treatment were increased. On the other hand, in S_1_ population WFY, DFY, Car, Tchl, CAT and POX activities improved by salinity memory (S_1t1_S_2_ and S_1t2_S_2_ treatments) compared to S_2_. Also, in S_1_ population, increases in DFYR, APX, R/S and R/SR were observed under S_1t2_S_2_ treatment. Therefore, it can be said that salinity memory was more evident in open-pollinated genotypes than corresponding selfed ones. Under secondary salinity stress condition with application of SA (H_2_S_2_), in OP population PH, DFY, PHR, CAT activity, RA (G), RA and RCL improved. Application of SA in severe salinity stress condition (H_2_S_2_) considerably improved DFY, DFYR, Tchl, APX, R/S and R/SR in selfed genotypes (Table 5). It can be said that the foliar spray of SA was effective in both S_1_ and OP genotypes. Results of the mean comparison of interaction effect of salinity treatments and pollination systems for indices indicated that salinity memory increased some spectral reflectance indices including WI, PSRI, ARI, GDVI and RGR in OP and WI, PSRI and RGR in S_1_ under S_1t1_S_2_ treatment compared to S_2_ treatment. Under S_1t2_S_2_ treatment enhanced NDVI, RARSa, RARSb, PSSR, SIPI, PSRI and RGR in both OP and S_1_ genotypes. Foliar spray of SA (1 mM) significantly improved NDVI, RARSa, RARSb, PSSR, SIPI, PSRI and RGR under severe salinity stress in both of OP and S_1_ compared to S_2_ treatment (Table 6). Overall, more spectral reflectance indices improved by salinity stress memory as well as application of salicylic acid despite the different pollination systems.

#### How did severe salinity stress influence genetic diversity in fescue

Genotypes tolerant to drought are not necessarily resistant to salinity stress. For example, severe salinity stress reduced RWC, WFY, DFYR, and R/S in sensitive genotypes, and also decreased Pro, RV(G), RA, RCL, and RDW in genotypes tolerant to drought (Table 7). While sensitive genotypes showed reductions in most indices including RARSb, SIPI, PSRI, CRI and GDVI compared to tolerant genotypes (Table 8).

**Table 7:** Mean comparison of salinity treatments (C, S_1t1_S_2_, S_1t2_S_2_, S_2_ and H_2_S_2_) separately for drought-sensitive and tolerant genotypes for some morphological, physiological and root traits.

**Morphological traits**												
**Treatment**	PH (cm)		RWC (%)		WFY (g/plant)		DFY (g/plant)		PHR (cm)		DFYR (g/plant)	
	**Sensitive**	**Tolerant**	**Sensitive**	**Tolerant**	**Sensitive**	**Tolerant**	**Sensitive**	**Tolerant**	**Sensitive**	**Tolerant**	**Sensitive**	**Tolerant**
**C**	12.75 ^bc^	11.93 ^bc^	50.28 ^bcd^	58.17 ^ab^	0.072 ^e^	0.124 ^ab^	0.041 ^e^	0.065 ^bc^	12.93 ^a^	11.31 ^ab^	0.128 ^b^	0.122 ^bc^
**S**_**1t1**_ **S**_**2**_	13.81 ^ab^	14.68 ^ab^	48.20 ^cd^	50.99 ^bcd^	0.087 ^cd^	0.137 ^a^	0.065 ^bc^	0.074 ^ab^	9.25 ^b^	8.75 ^b^	0.076 ^d^	0.080 ^d^
**S**_**1t2**_ **S**_**2**_	13.75 ^bc^	12.37 ^bc^	62.48 ^a^	50.47 ^bcd^	0.110 ^b^	0.089 ^cd^	0.076 ^ab^	0.052 ^de^	11.06 ^ab^	11.62 ^ab^	0.158 ^a^	0.109 ^c^
**S**_**2**_	11.37 ^c^	11.50 ^c^	46.73 ^d^	63.36 ^a^	0.079 ^de^	0.094 ^c^	0.049 ^de^	0.054 ^cd^	9.68 ^ab^	11.31 ^ab^	0.109 ^c^	0.131^b^
**H**_**2**_ **S**_**2**_	12.37 ^bc^	11.62 ^c^	56.99 ^abc^	47.91 ^d^	0.084 ^cde^	0.116 ^b^	0.078 ^a^	0.073 ^ab^	10.37 ^ab^	12.87 ^a^	0.109 ^c^	0.158 ^a^
Mean	12.81 ^A^	12.42 ^B^	52.94 ^A^	54.18 ^A^	0.086 ^B^	0.112 ^A^	0.062 ^B^	0.063 ^A^	10.66 ^A^	11.17 ^A^	0.116 ^B^	0.120 ^A^
**Physiological traits**												
**Treatment**	Car (mg/g leaf)		Tchl (mg/g leaf)		Pro (μmol/g leaf)		CAT (μmol min^-1^ mg^-1^ protein)		APX (μmol min^-1^ mg^-1^ protein)		POX (μmol min^-1^ mg^-1^ protein)	
	**Sensitive**	**Tolerant**	**Sensitive**	**Tolerant**	**Sensitive**	**Tolerant**	**Sensitive**	**Tolerant**	**Sensitive**	**Tolerant**	**Sensitive**	**Tolerant**
**C**	0.269 ^a^	0.234 ^abc^	1.03 ^a^	0.900 ^b^	1.79 ^bc^	1.51 ^c^	0.390 ^a^	0.203 ^c^	0.036 ^b^	0.026 ^cd^	0.189 ^bc^	0.137 ^de^
**S**_**1t1**_ **S**_**2**_	0.217 ^bcd^	0.190 ^cde^	0.847 ^bc^	0.723 ^cd^	1.86 ^abc^	1.91 ^abc^	0.368 ^a^	0.288 ^b^	0.061 ^a^	0.023 ^d^	0.406 ^a^	0.190 ^b^
**S**_**1t2**_ **S**_**2**_	0.252 ^ab^	0.214 ^bcd^	0.779 ^bc^	0.743 ^cd^	2.26 ^a^	1.55 ^c^	0.206 ^c^	0.188 ^c^	0.056 ^a^	0.031 ^bcd^	0.207 ^b^	0.159 ^cd^
**S**_**2**_	0.165 ^e^	0.196 ^cde^	0.463 ^f^	0.518 ^ef^	2.02 ^ab^	1.53 ^c^	0.135 ^d^	0.154 ^cd^	0.034 ^bc^	0.030 ^bcd^	0.152 ^d^	0.130 ^de^
**H**_**2**_ **S**_**2**_	0.174 ^de^	0.217 ^bcd^	0.473 ^f^	0.622 ^de^	1.83 ^abc^	1.53 ^c^	0.296 ^b^	0.341 ^ab^	0.056 ^a^	0.028 ^bcd^	0.146 ^de^	0.119 ^e^
Mean	0.215 ^A^	0.210 ^A^	0.720 ^A^	0.701 ^A^	1.95 ^A^	1.61 ^B^	0.279 ^A^	0.235 ^B^	0.048 ^A^	0.028 ^B^	0.220 ^A^	0.147 ^B^
**Root traits**												
**Treatment**	RV(G) (cm^3^/plant)		RA (cm^2^/plant)		RCL (cm/plant)		RDW (g/plant)		R/S		R/SR	
	**Sensitive**	**Tolerant**	**Sensitive**	**Tolerant**	**Sensitive**	**Tolerant**	**Sensitive**	**Tolerant**	**Sensitive**	**Tolerant**	**Sensitive**	**Tolerant**
**C**	0.390 ^bc^	0.235 ^e^	35.82 ^ab^	21.67 ^d^	321.7 ^ab^	197.2 ^d^	0.100 ^cd^	0.067 ^e^	3.28 ^b^	0.997 ^g^	0.699 ^ef^	0.405 ^f^
**S**_**1t1**_ **S**_**2**_	0.310 ^d^	0.414 ^ab^	32.37 ^bc^	37.75 ^a^	292.2 ^bc^	335.9 ^a^	0.115 ^bcd^	0.122 ^bc^	1.53 ^e^	1.98 ^d^	0.982 ^c^	1.71 ^b^
**S**_**1t2**_ **S**_**2**_	0.296 ^d^	0.301 ^d^	27.93 ^c^	27.97 ^c^	261.7 ^c^	258.2 ^c^	0.100 ^cd^	0.105 ^bcd^	1.35 ^ef^	4.21 ^a^	0.665 ^ef^	4.51 ^a^
**S**_**2**_	0.446 ^a^	0.345 ^cd^	40.06 ^a^	32.18 ^bc^	348.8 ^a^	295.6 ^bc^	0.153 ^a^	0.127 ^b^	1.22 ^fg^	3.08 ^b^	0.689 ^de^	0.883 ^cde^
**H**_**2**_ **S**_**2**_	0.213 ^e^	0.431 ^ab^	20.05 ^d^	39.27 ^a^	188.1 ^d^	349.5 ^a^	0.092 ^d^	0.158 ^a^	1.30 ^ef^	2.24 ^c^	0.817 ^cde^	0.946 ^cd^
Mean	0.331 ^B^	0.345 ^A^	31.25 ^A^	31.77 ^A^	282.5 ^A^	287.3 ^A^	0.112 ^B^	0.116 ^A^	1.74 ^B^	2.50 ^A^	0.764 ^B^	1.693 ^A^

Mean followed by the same letter is not significantly different according to LSD test (probability level of 5%).

PH, plant height; RWC, relative water content; WFY, wet forage yield; DFY, dry forage yield; PHR, plant height in recovery; DFYR, dry forage yield in recovery; Car, carotenoid content; Tchl, total chlorophyll; Pro, proline content; CAT, catalase activity; APX, ascorbate peroxidase activity; POX, peroxidase activity; RV(A), root volume (Archimedes); RA, root area; RCL, root cumulative length; RDW, root dry weight; R/S, root to shoot ratio; R/SR, root to shoot ratio in recovery.

**Table 8:** Mean comparison of salinity treatments (C, S_1t1_S_2_, S_1t2_S_2_, S_2_ and H_2_S_2_) separately for drought-sensitive and tolerant genotypes for some spectral reflectance indices.

**Spectral reflectance indices**												
**Treatment**	NDVI		SR		WI		RARSa		RARSb		PSSR	
	**Sensitive**	**Tolerant**	**Sensitive**	**Tolerant**	**Sensitive**	**Tolerant**	**Sensitive**	**Tolerant**	**Sensitive**	**Tolerant**	**Sensitive**	**Tolerant**
**C**	0.95 ^b^	0.86 ^cd^	21.48 ^a^	21.95 ^a^	0.853 ^c^	0.908 ^b^	0.074 ^e^	0.046 ^f^	7.05 ^de^	6.32 ^ef^	8.02 ^d^	6.56 ^e^
**S**_**1t1**_ **S**_**2**_	0.79 ^e^	0.82 ^de^	9.13 ^b^	10.79 ^b^	1.022 ^a^	1.000 ^a^	0.044 ^f^	0.046 ^f^	6.05 ^f^	6.69 ^def^	5.82 ^e^	6.35 ^e^
**S**_**1t2**_ **S**_**2**_	1.20 ^a^	1.24 ^a^	10.05 ^b^	10.34 ^b^	0.742 ^e^	0.758 ^de^	0.357 ^c^	0.266 ^d^	7.45 ^d^	13.46 ^b^	15.13 ^b^	16.50 ^a^
**S**_**2**_	0.90 ^bc^	0.94 ^b^	21.28 ^a^	26.76 ^a^	0.780 ^d^	0.767 ^de^	0.047 ^f^	0.049 ^f^	6.53 ^def^	10.79 ^c^	5.87 ^e^	6.72 ^e^
**H**_**2**_ **S**_**2**_	1.22 ^a^	1.19 ^a^	10.88 ^b^	13.52 ^b^	0.759 ^de^	0.773 ^d^	0.447 ^a^	0.371 ^b^	11.18 ^c^	14.86 ^a^	10.32 ^c^	14.95 ^b^
Mean	1.01 ^A^	1.01 ^A^	14.56 ^A^	16.67 ^A^	0.831 ^A^	0.841 ^A^	0.194 ^A^	0.155 ^B^	7.65 ^B^	10.42 ^A^	9.03 ^B^	10.22 ^A^
**Spectral reflectance indices**												
**Treatment**	SIPI		PSRI		CRI		ARI		GDVI		RGR	
	**Sensitive**	**Tolerant**	**Sensitive**	**Tolerant**	**Sensitive**	**Tolerant**	**Sensitive**	**Tolerant**	**Sensitive**	**Tolerant**	**Sensitive**	**Tolerant**
**C**	0.804 ^c^	0.674 ^d^	-0.080 ^d^	-0.115 ^e^	0.081 ^a^	0.026 ^f^	-0.024 ^b^	-0.024 ^b^	47.43 ^ab^	41.33 ^ef^	0.245 ^ef^	0.256 ^e^
**S**_**1t1**_ **S**_**2**_	0.659 ^d^	0.679 ^d^	-0.062 ^bc^	-0.067 ^cd^	0.028 ^ef^	0.027 ^ef^	-0.011 ^a^	-0.012 ^a^	45.34 ^c^	47.30 ^b^	0.487 ^cd^	0.459 ^d^
**S**_**1t2**_ **S**_**2**_	0.996 ^b^	1.11 ^a^	-0.057 ^bc^	-0.051 ^b^	0.064 ^b^	0.032 ^e^	-0.041 ^c^	-0.059 ^e^	40.29 ^f^	43.08 ^d^	0.520 ^b^	0.493 ^bc^
**S**_**2**_	0.631 ^d^	0.785 ^c^	-0.113 ^e^	-0.068 ^cd^	0.025 ^f^	0.056 ^c^	-0.020 ^b^	-0.020 ^b^	42.07 ^de^	48.72 ^a^	0.214 ^f^	0.160 ^g^
**H**_**2**_ **S**_**2**_	1.05 ^ab^	1.11 ^a^	-0.048 ^b^	-0.032 ^a^	0.044 ^d^	0.042 ^d^	-0.037 ^c^	-0.046 ^d^	42.07 ^de^	44.70 ^c^	0.476 ^cd^	0.585 ^a^
Mean	0.828 ^B^	0.873 ^A^	-0.072 ^A^	-0.066 ^A^	0.048 ^A^	0.036 ^B^	-0.026 ^A^	-0.032 ^B^	43.44 ^B^	45.02 ^A^	0.388 ^A^	0.390 ^A^

Mean followed by the same letter is not significantly different according to LSD test (probability level of 5%).

NDVI, normalized difference vegetation index; SR, simple ratio; WI, water index; RARSa, ratio analysis of reflectance spectra; RARSb, ratio analysis of reflectance spectra; PSSR, pigment specific simple ratio; SIPI, structure intensive pigment index; PSRI, plant senescence reflectance index; CRI, cartenoid reflectance index; ARI, anthocyanin reflectance index; GDVI, Green difference vegetation index; RGR, red/ green ratio.

#### What role did genetic diversity play in modulating the stress memory and application of SA in tall fescue

Under memory treatments (S_1t1_S_2_ and S_1t2_S_2_) DFY, Car, Tchl, CAT, APX and POX activities more enhanced in drought-sensitive genotypes compared to non-memory treatment (S_2_) (Table 7). While drought-tolerant genotypes had more Tchl and R/SR under S_1t1_S_2_ and S_1t2_S_2_ treatments. Thus, salinity stress memory was more pronounced in drought-sensitive genotypes of tall fescue. Foliar spray of SA in severe salinity stress condition significantly improved RWC, DFY, CAT and APX activities in drought-sensitive genotypes and WFY, DFY, DFYR, CAT, RV(G), RA, RCL and RDW in drought-tolerant genotypes (Table 7). The application of salicylic acid was more effective in reducing the effects of salinity stress in drought-tolerant genotypes. The response of spectral reflectance indices showed that WI, PSRI, ARI, GDVI and RGR significantly increased under S_1t1_S_2_ treatment compared to S_2_ treatment in sensitive genotypes and WI, ARI, and RGR in tolerant genotypes (Table 8). NDVI, RARSa, PSSR, SIPI, PSRI, and RGR enhanced in both drought-sensitive and tolerant genotypes under S_1t2_S_2_ treatment. Application of SA improved NDVI, RARSa, RARSb, PSSR, SIPI, PSRI and RGR in both drought-sensitive and tolerant genotypes under severe salinity stress (Table 8). Hence, stress memory and the foliar application of salicylic acid (SA) proved effective in mitigating the adverse effects of salinity stress across both drought-sensitive and tolerant genotypes, as indicated by various measured indices.

### Principal component analysis (PCA) and genotype selection

**Fig 4:**
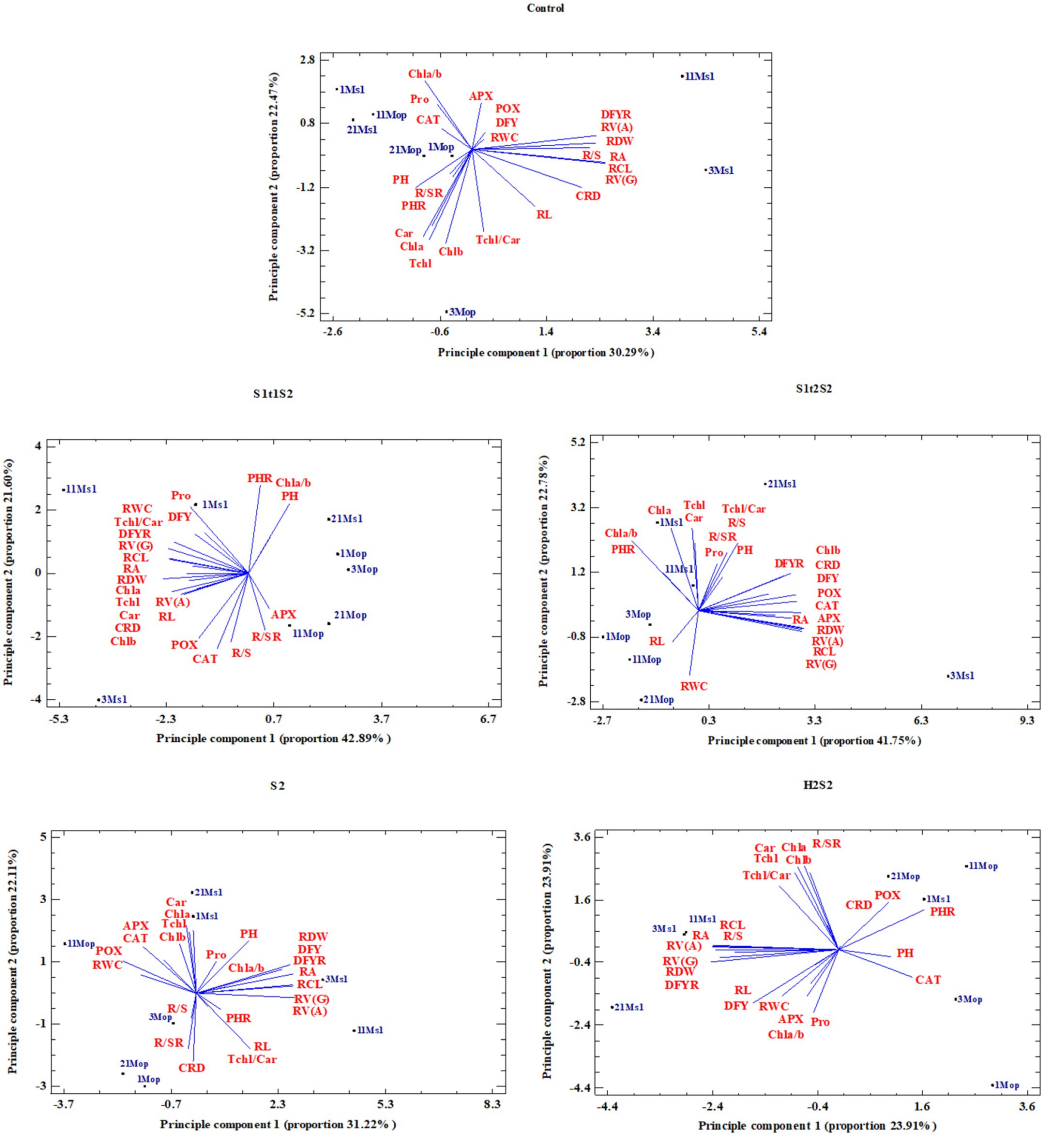
The biplot display of morphological, physiological, root traits and tall fescue genotypes in five salinity treatments (C, S_1t1_S_2_, S_1t2_S_2_, S_2_ and H_2_S_2_). CRD, crown diameter; PH, plant height; DFY, dry forage yield; PHR, plant height in recovery; DFYR, dry forage yield in recovery; RWC, relative water content; Chla, chlorophyll a content; Chlb, chlorophyll b content; Car, carotenoid content; Tchl, total chlorophyll; Chla/b, ratio of Chl*a*/Chl*b; Tchl/Car*, ratio of Tchl/Car; Pro, proline content; CAT, catalase activity; APX, ascorbate peroxidase activity; POX, peroxidase activity; RL, root length; RV(A), root volume (Archimedes); RV(G), root volume (giaroot); RA, root area; RCL, root cumulative length; RDW, root dry weight; R/S, root to shoot ratio; R/SR, root to shoot ratio in recovery.

To reduce the dimension of variables and drawing the association between characteristics and genotypes, principal component analysis (PCA) was used. Results of PCA revealed that the two first components explained more than 52, 53, 64, 64, and 58% of the variation in C, S_2_, S_1t1_S_2_, S_1t2_S_2_ and H_2_S_2_ treatments, respectively (S6 Table). In the control treatment, the first principal component (PC1) demonstrated stronger correlations with DFYR, CRD, RV(A), RV(G), RA, RCL, and RDW (S6 Table). Given that elevated values in these characteristics signify enhanced root growth capacity and, consequently, greater yield production for the genotypes, PC1 was designated as the ‘‘Root Characteristic System”. Principal component 2 (PC2) had negative correlations with Chla, Chlb, Car, Tchl, and Tchl/Car and a positive correlation with Chl a/b (S6 Table). Given that reduced values in these characteristics are indicative of higher photosynthetic capacity, PC2 was termed ‘‘Photosynthetic Capacity”. To categorize the genotypes using PCA, a biplot of PC1 and PC2 was created (Fig 4). Consequently, genotypes 11MS_1_ and 3MS_1_ exhibited a high potential for both root and yield production. Under S_2_ treatment, PC1 was positively correlated with DFY, DFYR, RV(A), RV(G), RA, RCL and RDW. Conversely, PC2 exhibited positive correlations with Chla, Car, and Tchl, while demonstrating negative correlations with CRD, RL, and R/SR (S6 Table). Genotypes 11MS_1_ and 3MS_1_ were hence identified as suitable genotypes for once severe salinity stress (Fig 4).

Under S_1t1_S_2_ treatment, PC1 had negative correlations with DFYR, RWC, RV(A), RV(G), RA, RCL and RDW. Consequently, selecting genotypes with low PC1 values can result in the identification of tolerant genotypes with a high root production capacity. Conversely, PC2 exhibited a positive association with PH and PHR, while showing a negative association with CAT and R/S (S6 Table). The biplot analysis of PC1 and PC2 (Fig 4) highlighted that genotypes 11MS_1_ and 1MS_1_, characterized by low PC1 and high PC2 values, were identified as preferable choices for first time pre-exposure salinity stress conditions. Under S_1t2_S_2_ treatment PC1 had positive correlations with DFY, DFYR, CAT, POX, RV(A), RV(G), RA, RCL and RDW. Therefore, selection based on high PC1 values can lead to tolerant genotypes with high potential for root production and yield. PC2 had positive correlations with Chla, Car, Tchl, Chl a/b, Tchl/Car, R/S and R/SR and a negative correlation with RWC (S6 Table). Therefore, genotypes with high PC2 values are well-suited for salinity stress conditions. As revealed by the biplot analysis of PC1 and PC2 (Fig 4), genotypes 21MS_1_ and 3MS_1_ were identified as particularly preferable choices for such conditions.Under H_2_S_2_ treatment, PC1 had high and negative correlations with DFYR, RV(A), RV(G), RA, RCL, RDW and R/S. PC2 had high and positive correlations with Chla, Chlb, Car, Tchl and Tchl/Car (S6 Table). Classify the genotypes based on PCA, genotypes 11MS_1_ and 3MS_1_ were found to have high potential to root production and yield (low PC1) and high photosynthetic capacity (high PC2) under the foliar spray of SA simultaneously with salinity stress (Fig 4).

### Correlation among indices and traits, and regression models

Correlation coefficients between spectral reflectance indices with different traits of tall fescue genotypes (Table 9) showed positive correlations between NDVI, RARSa and RARSb indices with CRD, RL, while negative correlations with Chla, Tchl, Chl a/b and Tchl/Car. Also, RARSb was positively correlated with R/S and R/SR. WI and NWI had positive correlations with PH, Chla, Chlb, Tchl, Chl a/b and Tchl/Car and a negative correlation with RL. PSSR had positive correlations with DFYR and RL. PSND, SIPI and PRI were positively correlated with CRD and RL, while negatively correlated with Tchl/Car. RNDVI, GNDVI and NDRE were significantly and negatively correlated with Chla, Tchl and Chl a/b but positively correlated with CRD and RL. CRI was positively correlated with CRD, DFYR, RL, RV(G), RA, RCL, RDW and R/S, while negatively correlated with Chl a/b. PSRI had positive correlations with CRD, PHR, RL, R/S and R/SR, but ARI had negative correlations with PHR, RL and R/SR and a positive correlation with Tchl/Car (Table 9).

**Table 9:** Correlation coefficients between spectral reflectance indices with different traits of tall fescue genotypes based on the average of two years for eight genotypes (1MOP, 1MS_1_, 3MOP, 3MPS_1_, 11MOP, 11MS_1_, 21MOP and 21MS_1_), five salinity treatments (C, S_1t1_S_2_, S_1t2_S_2_, S_2_ and H_2_S_2_) and two replications (n = 80).

Indices	NDVI	SR	WI	NWI	RARSa	RARSb	PSSR	PSND	SIPI	RNDVI	GNDVI	PRI	NDRE	PSRI	CRI	ARI	GDVI	RGR
Traits																		
**CRD**	0.23*	0.00	-0.19	-0.20	0.24*	0.34**	0.15	0.37**	0.35**	0.23*	0.33**	0.27*	0.22*	0.33**	0.38**	-0.20	-0.07	0.01
**PH**	-0.16	-0.20	0.29*	0.29*	-0.02	-0.18	-0.13	-0.13	-0.18	-0.16	0.04	0.02	0.02	0.05	-0.10	0.07	0.04	0.21
**DFY**	0.01	-0.02	0.17	0.17	0.01	0.07	0.21	0.06	0.02	0.02	0.20	-0.03	0.14	0.02	-0.09	0.14	0.07	0.10
**PHR**	0.20	0.23*	-0.09	-0.06	0.12	0.11	0.04	0.21	0.21	0.21	0.15	0.13	0.11	0.23*	-0.03	-0.28*	0.02	0.00
**DFYR**	0.10	0.09	-0.20	-0.20	-0.12	0.14	0.33**	0.13	0.11	0.11	0.18	-0.09	0.12	-0.03	0.37**	0.10	0.13	-0.08
**RWC**	0.09	0.26*	-0.12	-0.13	-0.10	0.29*	0.19	0.14	0.15	0.09	0.14	-0.04	0.09	0.02	0.10	0.02	0.09	-0.28*
**Chla**	-0.30**	-0.06	0.41**	0.42**	-0.28*	-0.32**	-0.03	-0.22*	-0.19	-0.31**	-0.33**	-0.18	-0.38**	-0.15	-0.05	0.10	0.09	0.06
**Chlb**	-0.11	-0.07	0.24*	0.27*	-0.17	-0.22*	-0.06	-0.12	-0.11	-0.11	-0.14	-0.09	-0.17	-0.20	0.16	0.03	-0.08	0.07
**Car**	-0.01	0.02	0.07	0.09	-0.10	-0.16	0.11	-0.03	-0.02	-0.01	-0.12	0.02	-0.17	-0.11	0.07	-0.05	-0.03	0.10
**Tchl**	-0.26*	-0.06	0.38**	0.40**	-0.26*	-0.31**	-0.04	-0.20	-0.18	-0.27*	-0.29*	-0.16	-0.34**	-0.17	0.01	0.09	0.05	0.06
**Chla/b**	-0.33**	-0.07	0.39**	0.35**	-0.22*	-0.25*	0.00	-0.21	-0.19	-0.34**	-0.30**	-0.17	-0.36**	0.02	-0.27*	0.17	0.29*	0.07
**Tchl/Car**	-0.47**	-0.14	0.62**	0.62**	-0.37**	-0.36**	-0.19	-0.35**	-0.32**	-0.48**	-0.40**	-0.33**	-0.43**	-0.19	-0.07	0.24*	0.11	0.04
**Pro**	-0.06	-0.01	0.11	0.06	0.09	-0.08	0.02	-0.09	-0.14	-0.06	0.01	0.14	0.03	-0.07	-0.06	0.08	0.03	0.06
**CAT**	0.04	-0.21	0.20	0.17	0.24*	-0.06	0.03	0.14	0.13	0.03	0.08	0.12	0.09	0.20	0.05	0.03	-0.08	0.20
**APX**	0.06	-0.07	0.05	0.03	-0.05	-0.11	0.17	0.02	-0.01	0.06	0.09	-0.10	0.04	-0.05	0.21	0.17	-0.14	0.06
**POX**	-0.15	-0.27*	0.27*	0.21	-0.08	-0.20	-0.17	-0.18	-0.19	-0.16	-0.15	-0.08	-0.16	-0.08	0.09	0.16	-0.20	0.14
**RL**	0.31**	-0.05	-0.23*	-0.25*	0.27*	0.27*	0.23*	0.40**	0.39**	0.30**	0.33**	0.29*	0.23*	0.31**	0.24*	-0.24*	-0.02	0.04
**RV(A)**	-0.02	-0.01	0.02	-0.01	-0.10	0.13	0.11	0.04	0.01	-0.02	0.14	-0.08	0.07	-0.02	0.21	0.12	0.09	-0.05
**RV(G)**	-0.07	0.02	0.04	0.00	-0.13	0.02	0.06	-0.05	-0.06	-0.07	0.00	-0.12	-0.04	-0.06	0.27*	0.08	0.15	-0.07
**RA**	-0.08	0.01	0.06	0.02	-0.14	0.01	0.06	-0.05	-0.07	-0.08	0.01	-0.13	-0.03	-0.05	0.27*	0.09	0.15	-0.06
**RCL**	-0.07	0.01	0.05	0.02	-0.13	0.01	0.07	-0.04	-0.05	-0.07	0.03	-0.12	-0.02	-0.05	0.27*	0.09	0.15	-0.06
**RDW**	-0.04	-0.02	-0.07	-0.11	-0.14	0.06	0.16	-0.05	-0.07	-0.03	0.05	-0.15	0.01	-0.07	0.23*	0.18	0.21	-0.03
**R/S**	0.12	0.08	-0.12	-0.10	-0.06	0.37**	0.17	0.21	0.19	0.12	0.23*	0.00	0.14	0.31**	0.46**	-0.12	0.19	-0.02
**R/SR**	0.17	-0.16	-0.03	-0.01	0.01	0.38**	0.16	0.21	0.17	0.17	0.24*	0.05	0.12	0.28*	0.00	-0.22*	-0.06	0.20

* and ** show significance at the 0.05 and 0.01 probability levels, respectively.

NDVI, normalized difference vegetation index; SR, simple ratio; WI, water index; NWI, normalized water index; RARSa, ratio analysis of reflectance spectra; RARSb, ratio analysis of reflectance spectra; PSSR, pigment specific simple ratio; PSND, pigment specific normalized different; SIPI, structure intensive pigment index; RNDVI, red normalized difference vegetation index; GNDVI, green normalized difference vegetation index; PRI, photochemical reflectance index; NDRE, normalized difference red edge index; PSRI, plant senescence reflectance index; CRI, cartenoid reflectance index; ARI, anthocyanin reflectance index; GDVI, green difference vegetation index; RGR, red/ green ratio.

CRD, crown diameter; PH, plant height; DFY, dry forage yield; PHR, plant height in recovery; DFYR, dry forage yield in recovery; RWC, relative water content; Chla, chlorophyll a content; Chlb, chlorophyll b content; Car, carotenoid content; Tchl, total chlorophyll; Chla/b, ratio of Chla/Chlb; Tchl/Car, ratio of Tchl/Car; Pro, proline content; CAT, catalase activity; APX, ascorbate peroxidase activity; POX, peroxidase activity; RL, root length; RV(A), root volume (Archimedes); RV(G), root volume (Giaroot); RA, root area; RCL, root cumulative length; RDW, root dry weight; R/S, root to shoot ratio; R/SR, root to shoot ratio in recovery.

Stepwise multiple linear regression (Table 10) indicated that spectral indices of CRI could be considered as independent variable that explained most of the ratio of root/shoot variation (r = 0.46). RARSb and ARI explained some variation of relative water content (RWC) (r = 0.43). WI and RARSa made a significant multivariate regression model with CAT activity (r = 0.42). The result of stepwise regression also indicated plant height in recovery can explain by SR and ARI (r = 0.40). PSSR and CRI could be regarded as independent variables that account for a portion of the variation in dry forage yield in recovery (r = 0.46) (Table 10).

**Table 10:** Results from stepwise regression analysis for predicting some measured traits by spectral reflectance indices in eight tall fescue genotypes evaluated under five salinity treatments (C, S_1t1_S_2_, S_1t2_S_2_, S_2_ and H_2_S_2_) based on the average of two years.

Dependent variable	Equation	R
PH (cm)	**Y** = 4.23 + 8.21 **WI** + 3.87 **RGR**	0.36
DFY (g/plant)	**Y** = - 0.045+ 0.105 **WI** + 0.002 **PSSR**	0.36
PHR (cm)	**Y** = 7.25 + 0.121 **SR** − 59.01 **ARI**	0.40
DFYR (g/plant)	**Y** = 0.044 + 0.004 **PSSR** + 0.790 **CRI**	0.46
RWC (%)	**Y** = 54.25 + 1.02 **RARSb** − 25.57 **ARI**	0.43
Car (mg/g leaf)	-	-
Tchl (mg/g leaf)	**Y** = 0.88 + 1.72 **NWI**	0.39
Pro (μmol/g leaf)	-	-
CAT (μmol min^-1^ mg^-1^ protein)	**Y** = - 0.38 + 0.69 **WI** + 0.34 **RARSa**	0.42
APX (μmol min^-1^ mg^-1^ protein)	**Y** = 0.031 + 0.16 **CRI**	0.20
POX (μmol min^-1^ mg^-1^ protein)	**Y** = -2.21 + 2.39 **WI** − 4.07 **NWI**	0.36
RV(G) (cm^3^/plant)	**Y** = 0.23 + 2.37 **CRI**	0.27
RA (cm^2^/plant)	**Y** = 22.34 + 215.06 **CRI**	0.26
RCL (cm/plant)	**Y** = 201.68 + 1951.68 **CRI**	0.27
RDW (g/plant)	**Y** = 0.094 + 0.460 **CRI**	0.22
R/S	**Y** = 0.98 + 26.67 **CRI**	0.46
R/SR	**Y** = - 0.25 + 0.163 **RARSb**	0.38

PH, plant height; DFY, dry forage yield; PHR, plant height in recovery; DFYR, dry forage yield in recovery; RWC, relative water content; Car, carotenoid content; Tchl, total chlorophyll; Pro, proline content; CAT, catalase activity; APX, ascorbate peroxidase activity; POX, peroxidase activity; RV(G), root volume (Giaroot); RA, root area; RCL, root cumulative length; RDW, root dry weight; R/S, root to shoot ratio; R/SR, root to shoot ratio in recovery.

NDVI, normalized difference vegetation index; SR, simple ratio; WI, water index; NWI, normalized water index; RARSa, ratio analysis of reflectance spectra; RARSb, ratio analysis of reflectance spectra; PSSR, pigment specific simple ratio; PSND, pigment specific normalized different; SIPI, structure intensive pigment index; RNDVI, red normalized difference vegetation index; GNDVI, green normalized difference vegetation index; PRI, photochemical reflectance index; NDRE, normalized difference red edge index; PSRI, plant senescence reflectance index; CRI, cartenoid reflectance index; ARI, anthocyanin reflectance index; GDVI, green difference vegetation index; RGR, red/ green ratio.

## Discussion

### The effect of severe salinity stress and pollination system

Salinity, heat, and water deficit are the most abiotic stresses which cause significant damage on plants [49]. Stress memory, which occurs in some plants, plays an important role in adaptation to these adverse conditions [50]. When subjected to salt stress, plants, as sessile organisms, must initiate responses and adaptations to counter adverse conditions. Consequently, delving into the mechanisms of salt stress response and discovering strategies to enhance plant stress tolerance becomes imperative [51]. Changes in canopy characteristics, including leaf chlorophyll, other photosynthetic pigment contents, dry matter, and plant water status, have been observed to alter in response to the ionic and osmotic components of salinity stress. These changes result in noticeable variability in the spectral reflectance of the canopy, particularly in the visible (VIS) and near-infrared (NIR) domains [52–54]. Different spectral reflectance indices (SRIs) have been formulated as alternative means of selection in breeding trials, replacing the need for destructive and direct selection criteria [55]. A solution to deal with salt stress is using plant growth regulators.

Plant hormones, recognized as a sustainable tool aid in modifying the detrimental repercussions of both abiotic and biotic strains on plants. Salicylic acid a phenolic compound synthesized by root cells in numerous plants, governs physiological processes and diminishes the repercussions of stress, thereby enhancing the harmful consequences of salinity via signaling mechanisms [56]. In this study we evaluated the consequences of salinity memory during two different stages and foliar application of SA under salinity stress compared with a single salinity on two selfed and open-pollinated reproductive systems of tall fescue. Considerable genotypic variation observed among genotypes, pollination systems and salinity treatments in terms of most morphological, physiological, root traits and spectral indices which can facilitate the selection process in this plant.

No inbreeding depression was observed for biomass, photosynthetic capacity, enzymatic antioxidant activities (e.g., APX and POX), and potential of root production which can be attributed to the low heterosis for these traits. This facilitates the development of inbred lines in this open-pollinated plant for further studies. The enhancement of these traits through selfing suggests that associated additive genes become fixed in the selfing system. Consequently, methods founded on recurrent selection may propose an increased response to selection for these traits in tall fescue. Geiger [57] and Waser and Price [58] reported that significant outbreeding depression is sometimes observed in crosses between distant populations of the same species. However, Saeidnia *et al*. [10] reported that the S_1_ families exhibited lower performance compared to the OP ones. The stage-specific variation in inbreeding depression could be linked to genes expressed at different stages [59].

In the present study, once severe salinity stress treatment (S_2_) decreased PH, WFY, DFY, PHR, WFYR, DFYR, Chla, Chlb, Car, Tchl, Chl a/b, TChl/Car, CAT and POX activities. Initially, salt stress causes water loss and growth inhibition, which constitutes the ion-independent response. As salt stress persists, Na^+^ accumulates to toxic concentrations, leading to premature senescence, known as the ion-dependent response [60, 61]. Fan *et al*. [51] noted that salt stress adversely affected ion homeostasis in bermudagrass cells, potentially leading to negative impacts on the plant’s growth and the absorption of nutrient elements [62]. A comparable outcome was reported in Arabidopsis under osmotic stress [63], aligning with our findings. Photosynthesis is pivotal for plants, and its efficiency is sensitive to stress conditions [64]. Chlorophyll, as a fundamental component of photosynthesis, directly influences the plant’s photosynthetic efficiency. Salt stress is known to reduce chlorophyll synthesis and increase chlorophyllase-mediated degradation [65]. Fan *et al*. [51] showed that the Chl a/b ratio decreased in bermudagrass following salt stress treatment. Consistent with our results, Hu *et al*. [66] reported that the salt stress reduced Chla and Chlb in perennial ryegrass. Abiotic stress could also lead to an overproduction of ROS, resulting in lipid peroxidation and harm to proteins and nucleic acids [66–68]. The findings imply that salt stress has the potential to induce an excess of ROS, and that the function of CAT and POX in eliminating ROS may be significant. However, some studies including Hu *et al*. [69] reported that the activity of antioxidant enzymes such as APX, POX, CAT and SOD increased in some *Lolium perenne* L. cultivars after four days’ treatment with 250 mM NaCl. Hence, the impact of stresses on enzyme activity is contingent on the crop species, the type of stress, and the duration and intensity of the stress.

Once severe salinity stress (S_2_) increased CRD, Pro and all root characteristics. The accumulation of proline is recognized to be linked with stress conditions, particularly those involving osmotic stress and high-temperature stress [70, 71]. Li *et al*. [72] expressed that the NaCl stress resulted in an increase of Pro content in *Festuca valesiaca* and *Festuca glauca* which was in agreement with our results. Water absorption efficiency by plant roots is a vital determinant of salinity resistance, and water absorption relies on the root size and its spatial distribution [73]. Different studies have been conducted on root characteristic system [74–78], whereas the role of salinity, on tall fescue root system growth and mass is still unclear. Hameed and Ashraf (2008) [79] demonstrated that under saline conditions (200 mM NaCl), the grass population originating from saline soil exhibited higher fresh and dry biomass of roots compared to the population from non-saline conditions. This finding is in agreement with the results obtained in this study. The increase in root mass to shoot mass ratio is a frequent reaction to salt stress which was also shown in this study. The increase in root-to-shoot ratio (RSR) is associated with features related to water stress and osmotic effects rather than direct salinity effects. In the context of salinity stress, a larger root mass can facilitate the retention of toxic ions in root cellular tissues and regulate their translocation to plant organs, such as the stem and leaves. This characteristic mechanism contributes to the plant’s resistance to salinity.

The results showed that under severe salinity stress, open-pollinated genotypes displayed negative effects on certain traits, while in sensitive genotypes, the stress effect also manifested in some traits and indices. The impact of salinity stress on pollination systems and genetic diversity in tall fescue highlighted that selecting and breeding salinity-tolerant genotypes can play a crucial role in enhancing plant resilience, preserving genetic diversity, and maintaining reproductive efficiency in saline environments.

### Consequence of salinity memory

Recent publications indicated that the plants pre-exposed to abiotic stresses can achieve the potential to display a stronger and faster activation of their defense system in response to the subsequent stress challenges as “stress memory” [80, 81]. Short-term stress memory predominantly results from temporary alternation in physiological and biochemical metabolites. Once the stress subsides, plants revert to their previous growth status, essentially "forgetting" the stress event [82]. In contrast, long-term stress memory, governed by epigenetic mechanisms, has the potential to endure throughout the entire life of the plant that experienced the stress and may even be transmitted to its offspring [83]. In the current research we applied stress memory in two different stages (45 and 90 days after establishment, S_1t1_S_2_ and S_1t2_S_2_, respectively) to see how the stage of applying affects this phenomenon. The results showed that similar trends were observed in both stages in terms of measured traits with the exception of RWC, PHR, WFYR, DFYR, RL and R/S. Both S_1t1_S_2_ and S_1t2_S_2_ induced stress memory by increasing PH, WFY, DFY, all physiological traits (including photosynthesis pigments, proline content and activity of enzymes) and R/SR compared to S_2_ treatment. When plants experience pre-exposure to stress, they can generate stable signals and physiological responses that may persist as "stress memory" [78]. These results was consistent with previous studies that have shown similar memory effects in other plant species under different stress conditions [10, 18, 21, 50, 84]. Hu *et al*. [85] reported that multiple and short time salt stress/recovery treatments, resulted to salt stress memory in ryegrass. Hu *et al*. [85] showed that higher H_2_O_2_ and O_2_^−^ accumulation in root hair and higher leaves electrolyte leakage level in control plants than in pre-treated. Higher accumulation of H_2_O_2_ might be attributed to the super transcriptional abundance of CAT in treated root, which was consistent with our findings in relation to CAT activity measured in leaves. Hu *et al*. [78] documented that pre-acclimation treatment inhibited the decline at various steps of OJIP (Chl a fluorescence induction transient) and energy transport fluxes in the active Photosystem II reaction center (PSII RC) for tall fescue genotypes. The plant response to abiotic stress involves alterations in the accumulation of various metabolites. Increased or decreased production of certain metabolites can contribute to enhanced tolerance to abiotic stress in plants [86–88], which was examined as a potential factor in stress memory responses. Furthermore, there are reports indicating that heat stress memory correlates with notable shifts in leaf metabolite profiles [78].

Our results suggest that the combined analysis of different stress stages, pollination systems, and genetic diversity provided a comprehensive view of salt stress memory in tall fescue. In particular, we found that interactions between these factors significantly influence mechanisms of salt stress response and highlight the importance of testing multiple factors simultaneously. For example, the results of the interaction effect of salinity treatments and pollination systems showed that salinity memory was more evident in open-pollinated than selfed in terms of traits. Also, salinity stress memory was more pronounced in drought-sensitive genotypes. The improved performance in drought-sensitive genotypes indicated a potential for using salinity memory as a strategy to enhance stress tolerance in more vulnerable plants. Mendanha *et al*. [89] and Amini *et al*. [84] in wheat stated that less productive genotypes (in terms of grain not biomass) had better response to stress memory which was in agreement with our reports. These findings highlights the need for a multifaceted approach to studying plant stress responses, beyond a single analysis to better understand complex interactions.

### Effects of salicylic acid

In the current study, spraying tall fescue genotypes with SA under severe salinity stress treatment (H_2_S_2_) caused enhancement PH, WFY, DFY, PHR, DFYR, most photosynthesis pigments (including Chlb, Car, Tchl and Tchl/Car), CAT, APX activities, RL and R/SR compared with untreated plants under the same salinity stress condition (S_2_). Our findings that foliar application of salicylic acid (SA) mitigates the effects of salinity stress was in line with previous research by Kentucky bluegrass and tall fescue, which demonstrated improved stress tolerance with SA application [28, 37]. This supports the hypothesis that SA plays a crucial role in enhancing stress responses in plants. Given that salt stress inhibits plant growth by negatively impacting several physiological and biochemical processes such as photosynthesis, antioxidant capacity, and ion homeostasis [90], it is proposed that the observed improvement in growth of salt-stressed plants following SA treatment could be attributed to SA-induced alterations in these processes. For instance, El-Tayeb [91] demonstrated that the SA-induced growth enhancement might be attributed to the increase activity of antioxidants. Consequently, photosynthesis, a pivotal determinant of plant growth and yield [92], could have been augmented due to SA application, consistent with the findings of this investigation. SA application was also shown to boost photosynthetic rates in various crops, such as barley [93], soybean [94], wheat [95], and maize [96]. The results of the interaction effect of salinity treatments and pollination systems represented the application of salicylic acid was equally effective in reducing the effects of salinity stress in both S_1_ and OP. The foliar spray of SA was more effective in tolerant genotypes compared to sensitive genotypes in terms of evaluated traits.

### Selection of superior genotypes

In the once severe salinity stress condition (S_2_), PC1 showed positive correlations with root traits, DFY, and DFYR, while PC2 exhibited positive correlations with Chla, Car, and Tchl. Genotypes 11MS_1_ and 3MS_1_, characterized by high PC1 and low PC2 values, were identified as preferable for once severe salinity stress conditions. These genotypes displayed an extensive root system and higher yield but lower photosynthetic capacity.

Under the S_1t1_S_2_ treatment, PC1 showed negative correlations with root traits. PC2 exhibited positive correlations with PH and PHR. Low PC1 indicated an extensive root system. High PC2 indicated greater yield production capacity. Therefore, genotypes 11MS_1_ and 1MS_1_ (low PC1, high PC2) were identified as preferable for first time pre-exposure to salinity stress. Genotypes 11MOP and 21MOP (susceptible) had less extensive root systems and lower yields. Under S_1t2_S_2_ treatment, PC1 had positive relevance with DFY, DFYR, CAT, POX activities and root characteristics and PC2 had positive correlations with photosynthetic pigments. Hence genotype 21MS_1_ (with high PC1 and PC2) was identified as tolerant genotype for this condition. Under H_2_S_2_ treatment, PC1 had negative correlations with DFYR and root characteristics and PC2 had positive correlations with photosynthetic pigments. Therefore, genotypes 11MS_1_ and 3MS_1_ (with low PC1 and high PC2) were identified as genotypes that positively responded to the application of salicylic acid to reduce the effect of severe salinity stress.

In general, genotype 11M was identified as a suitable genotype not only in respect to stress memory potential (for first time pre-exposure salinity stress) but also as a salinity-tolerant genotype (for once severe salinity stress conditions). Genotypes 11MS_1_ and 3MS_1_ were also found to have a high potential to root and photosynthetic capacity under the foliar spray of SA simultaneously with salinity stress. Saeidnia *et al*. [7, 10] reported that principal component analysis is a powerful multivariate method for identifying superior genotypes when a complex set of variables have been measured.

### Spectral reflectance indices (SRIs)

Two types of spectral reflectance indices (SRIs) provide alternative indirect selection tools for descriptive traits: vegetation-SRIs and water-SRIs. The former is composed of various wavelengths in the visible (VIS: 400–700 nm) and red-edge (NIR: 700–850 nm) regions [55]. Recent reports indicate that the wavelengths of 420–470 nm (blue region) effectively characterizes the absorption features of photosynthetic parameters and have been recognized as regions sensitive to salinity stress in many crops [97–99]. The current research results showed that S_2_ treatment reduced SRIs including WI, RARSa, PSSR, CRI, and RGR compared with control treatment. There have been limited studies investigating the use of spectral reflectance indices (SRIs) for detecting variations in phenotypic parameters under saline conditions. Reduced chlorophyll content in stressed plants results in diminished chlorophyll absorption, which manifests as declining reflectance values in the red-edge zone of the spectra [100] which was consistent with our results regarding some indices. The decline in chlorophyll levels may stem from the interference of Na^+^ and Cl^-^ ions with enzymes involved in chlorophyll biosynthesis or from disruptions in the integration of chlorophyll molecules into stable complexes [101]. Water index (WI) has been demonstrated to predict relative water content, leaf water potential, stomatal conductance, and canopy temperature with sufficient water stress [102]. Peñuelas *et al*. [103] showed the usefulness of using WI to assess the effect of salinity on barley. Gao and Li [104] demonstrated that salinity stress in tall fescue can be reflected by parameters at various levels of physiological processes. However, these parameters exhibit uneven sensitivity, making them less suitable as indicators for salinity tolerance selection in breeding. In this study, at least three indices calculated from single leaf reflectance spectrum (mSR750/705, mND750/705, and SI710/760) were identified as sensitive for differentiating salinity stress from untreated plants. It is noted that some wavelengths used in the SRIs that yielded significant results in prior studies may not be universally applicable across all environmental conditions. The lack of a universal relationship between published SRIs and measured parameters may stem from differences in crop types, growth stages, and levels of stress [29].

Foliar application of SA and stress memory alleviated the damage of salinity stress by promoting physiological characteristics which can determine through the function spectral reflectance indices. Our results showed that genotypes (G), pollination systems (P) and their interaction with five salinity treatments (T) had significant effects on most SRIs emphasizing the possibility of selection of genotypes with the positive response to salinity tolerance in this germplasm. In the present study, under S_1t1_S_2_ treatment enhanced WI and some vegetation-SRIs in OP and WI, PSRI and RGR in S_1_ and under S_1t2_S_2_ treatment increased most vegetation-SRIs in both OP and S_1_. Under H_2_S_2_ treatment improved most vegetation-SRIs in both OP and S_1_. So, application of salicylic acid and salinity memory in both OP and S_1_ genotypes were equally effective in reducing the harmful effects of salinity stress by improving some spectral reflectance indices. Under S_1t1_S_2_ treatment improved WI, PSRI, ARI, GDVI and RGR in sensitive genotypes and WI, ARI and RGR in tolerant genotypes. Under S_1t2_S_2_ treatment enhanced most vegetation-SRIs in both sensitive and tolerant genotypes. Under H_2_S_2_ treatment increased most vegetation-SRIs in both sensitive and tolerant genotypes. Thus, salinity memory and foliar application of SA were effective in order to reduce the harmful effects of salinity stress in both sensitive and tolerant genotypes by improving some spectral reflectance indices.

### Correlation between SRIs with traits and stepwise multiple linear regression (SMLR)

Recent advancements in high-throughput tools have empowered plant breeders, allowing them to accelerate the pace of genetic gain by thoroughly evaluating traits that were traditionally challenging to assess rapidly and nondestructively. The present study explores the potential utilization of spectral reflectance indices as alternative selection tools for traits typically measured destructively in tall fescue. Results of phenotypic correlation coefficients between spectral reflectance indices with different traits revealed that WI and NWI had significantly positive correlations with PH and photosynthetic pigments (Chla, Chlb, Tchl, Chl a/b and Tchl/Car). While some vegetation-SRIs (including NDVI, RARSa, RARSb and others) had negative correlations with some photosynthetic pigments. According to these results, high values of WI and NWI and low values NDVI, RARSa and RARSb will lead to select genotypes with higher photosynthetic capacity. We also were found positive relationships between CRI with root traits, CRD and DFYR. Various investigations have highlighted a noteworthy association between spectral characteristics and plant biomass, along with leaf area index, in crops such as eggplant (*Solanum melongena* L.) [105] and seashore paspalum (*Paspalum vaginatum* Swartz) [33] when grown in saline soils.

To identify the most influential spectral reflectance indices (SRIs) explaining variability in each destructively measured trait across genotypes, we employed stepwise multiple linear regression (SMLR) with all SRIs as independent variables. The SMLR model identified the Chlorophyll Reflectance Index (CRI) from vegetation-SRIs as the most influential SRI explaining much of the variation in the root/shoot ratio. For dry forage yield in recovery, indices from vegetation-SRIs (PSSR and CRI) exhibited high explanatory power, while combined indices from both vegetation-SRIs and water-SRIs were the best predictors of Catalase (CAT) activity. In stepwise regression for relative water content, RARSb and ARI, and for plant height in recovery, SR and ARI were identified as the most informative indices. This indicates that vegetation-specific spectral reflectance indices (SRIs), which combine data from the blue, green, and red-edge spectral regions are suitable for assessing similar vegetation-related characteristics across the crop canopy. Conversely, indices derived from water-SRIs, which integrate information from NIR and SWIR regions, act as supplementary tools to vegetation-SRIs, enabling the evaluation of plant traits associated with alterations in plant water status and internal leaf structure. Earlier research has underscored the efficacy of SRIs that amalgamate data from the blue, green, red, and red-edge wavebands in phenotyping diverse vegetation parameters, encompassing above-ground biomass and morphological traits linked to growth status [106, 107]. On the contrary, indices that merge information from NIR and SWIR wavebands prove effective in indirectly estimating yield, water content, and various plant physiological characteristics [108–110].

Despite the comprehensive analysis performed in this study, several limitations should be acknowledged. First, the experiments were conducted under controlled greenhouse conditions, which may not fully replicate field conditions. Second, the genetic diversity of the tall fescue genotypes used in this study was only four, which may not represent the genetic variability of the species. Future studies should include different genotypes and conduct field trials to confirm these findings under natural conditions.

## Conclusions

The findings of this study suggest that salinity stress can significantly impact the morphological, root, and physiological functions of tall fescue genotypes, consequently affecting plant growth and biomass production. Therefore, selecting and breeding salinity-tolerant genotypes can play a crucial role in enhancing plant resilience, preserving genetic diversity, and maintaining reproductive efficiency in saline environments. The significant genetic variation between pollination systems (selfed and open-pollinated) indicated that any changes in plant natural mating systems could clearly change the germplasm genetic structure. Pre-exposed to an early salinity caused significant results in the subsequent plant stages with the emphasis of physiological traits. The results showed that similar trends were observed in both stages of pre-exposure salinity in terms of measured traits with the exception of RWC, PHR, WFYR, DFYR, RL and R/S. However, salinity memory was more evident in open-pollinated than selfed population. Additionally, salinity memory was more pronounced in drought-sensitive genotypes of tall fescue, allowing them to exhibit a faster and more protective response to periodic salinities. Foliar application of salicylic acid improved tall fescue genotype’s tolerance to salinity stress by promoting yield productivity, photosynthesis pigments (Chla, Chlb and Tchl), non-enzyme (e.g., Car), enzymatic antioxidant (e.g., CAT and APX) activities, root length and ratio of root/shoot in recovery. The results of PCA indicated the superiority of genotype 11M under most salinity treatments and can be used in the development of synthetic varieties and further breeding programs. Comparing various spectral reflectance indices demonstrated vegetation-SRIs (RARSa, PSSR and RGR) were sensitive to explain important traits. Further research is required to validate these indices across various genotypes and physiological attributes, establishing them as a model for the rapid screening of salinity-tolerant materials in breeding projects.

## Supporting information

S1 Fig Different stages for mesuring various traits (morphological, physiological and root) in five salinity treatments (C, S_1t1_S_2_, S_1t2_S_2_, S_2_ and H_2_S_2_) in tall fescue genotypes. (DOCX)

S2 Fig Mean comparison emergence rate for the interactions of four tall fescue genotypes (1M, 3M, 11M and 21M) and two different pollination systems (selfed (S_1_) and open-pollinated (OP)) before salinity stress during two years. Mean followed by the same letter is not significantly different according to LSD test (probability level of 5%).
(DOCX)

S3 Fig Mean comparison of relative water content for the interaction of two different pollination systems (selfed (S1) and open-pollinated (OP)) and two salinity treatments (C and S1) in the first time (a) and in the second time (b) during two years. Mean comparison total chlorophyll for the interaction of two different pollination systems (selfed (S1) and open-pollinated (OP)) and two salinity treatments (C and S1) in the first time (c) and in the second time (d) during two years. Mean comparison of proline content for the interaction of two different pollination systems (selfed (S1) and open-pollinated (OP)) and two salinity treatments (C and S1) in the first time (e) and in the second time (f) during two years. Mean followed by the same letter is not significantly different according to LSD test (probability level of 5%).
(DOCX)

S1 Table The formula of different spectral reflectance indices, which were used in this study. (DOCX)

S2 Table Mean squares of morphological traits and relative water content in four tall fescue genotypes and two different pollination systems (selfed (S_1_) and open-pollinated (OP)) in five salinity treatments (C, S_1t1_S_2_, S_1t2_S_2_, S_2_ and H_2_S_2_) evaluated during two years. (DOCX)

S3 Table Mean squares of physiological traits in four tall fescue genotypes and two different pollination systems (selfed (S_1_) and open-pollinated (OP)) in five salinity treatments (C, S_1t1_S_2_, S_1t2_S_2_, S_2_ and H_2_S_2_) evaluated two years. (DOCX)

S4 Table Mean squares of root traits in four tall fescue genotypes and two different pollination systems (selfed (S_1_) and open-pollinated (OP)) in five salinity treatments (C, S_1t1_S_2_, S_1t2_S_2_, S_2_ and H_2_S_2_) evaluated during two years. (DOCX)

S5 Table Correlation coefficients among spectral reflectance indices based on the average of two years for eight genotypes (1MOP, 1MS_1_, 3MOP, 3MPS_1_, 11MOP, 11MS_1_, 21MOP and 21MS_1_), five salinity treatments (C, S_1t1_S_2_, S_1t2_S_2_, S_2_ and H_2_S_2_) and two replications (n = 80). (DOCX)

S6 Table The first two principal components (PC) loadings for the measured traits on 8 tall fescue genotypes in five treatment levels (C, S_1t1_S_2_, S_1t2_S_2_, S_2_ and H_2_S_2_) during two years. (DOCX)

## Abbreviations

APX: ascorbate peroxidase activity
ARI: anthocyanin reflectance index
C: control treatment
Car: carotenoid content
CAT: catalase activity
Chl a/b: ratio of Chla/Chlb
Chla: chlorophyll a content
Chlb: chlorophyll b content
CRD: crown diameter
CRI: cartenoid reflectance index
DFY: dry forage yield
DFYR: dry forage yield in recovery
G: genotypes
GDVI: green difference vegetation index
GNDVI: green normalized difference vegetation index
H_2_S_2_: foliar spray of SA simultaneously with secondary salinity stress
NDRE: normalized difference red edge index
NDVI: normalized difference vegetation index
NWI: normalized water index
OP: open-pollinated
P: pollination system
PH: plant height
PHR: plant height in recovery
POX: peroxidase activity
PRI: photochemical reflectance index
Pro: proline content
PSND: pigment specific normalized different
PSRI: plant senescence reflectance index
PSSR: pigment specific simple ratio
R/S: root to shoot ratio
R/SR: root to shoot ratio in recovery
RA: root area
RARSa: ratio analysis of reflectance spectra
RARSb: ratio analysis of reflectance spectra
RCL: root cumulative length
RDW: root dry weight
RGR: red/ green ratio
RL: root length
RNDVI: red normalized difference vegetation index
RV(A): root volume (Archimedes)
RV(G): root volume (Giaroot)
RWC: relative water content
RWW: root wet weight
S_1_: selfed
S_1t1_: primary mild salinity stress in first time
S_1t1_S_2_: twice salinity stress treatment (primary in the first time and secondary at the end stage)
S_1t2_: primary mild salinity stress in second time
S_1t2_S_2_: twice salinity stress treatment (primary in the second time and secondary at the end stage)
S_2_: once severe salinity stress treatment (secondary only)
SIPI: structure intensive pigment index
SR: simple ratio
SRIs: spectral reflectance indices
T: treatment
Tchl/Car: ratio of Tchl/Car
Tchl: total chlorophyll
WFY: wet forage yield
WFYR: wet forage yield in recovery
WI: water index
