# Supplementary material for: Salt stress memory in tall fescue: Interaction of different stress stages, pollination system and genetic diversity
Source: PLoS One. 2024 Sep 12;19(9):e0310061. doi: 10.1371/journal.pone.0310061 (PMC11392345; doi:10.1371/journal.pone.0310061)
Supplement: S1 Fig — (DOCX) [file pone.0310061.s001.docx]

| 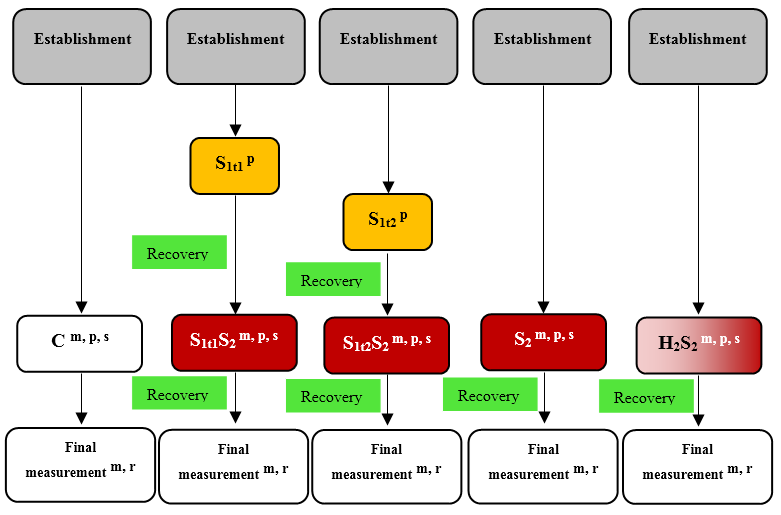 |
| --- |
| **S1 Fig. Different stages for mesuring various traits (morphological, physiological and root) in five salinity treatments (C, S_1t1_S_2_, S_1t2_S_2_, S_2_ and H_2_S_2_) in tall fescue genotypes.**  **^m^ - Time of morphological traits measurement, ^p^ - Time of physiological traits measurement, ^r^ - Time of root traits measurement, ^s^ - Time of spectral reflectance measurement** |
