# Supplementary material for: Salt stress memory in tall fescue: Interaction of different stress stages, pollination system and genetic diversity
Source: PLoS One. 2024 Sep 12;19(9):e0310061. doi: 10.1371/journal.pone.0310061 (PMC11392345; doi:10.1371/journal.pone.0310061)
Supplement: S2 Fig — Mean followed by the same letter is not significantly different according to LSD test (probability level of 5%). (DOCX) [file pone.0310061.s002.docx]

|  |
| --- |
| **S2 Fig. Mean comparison emergence rate for the interactions of four tall fescue genotypes (1M, 3M, 11M and 21M) and two different pollination systems (selfed (S_1_) and open-pollinated (OP)) before salinity stress during two years. Mean followed by the same letter is not significantly different according to LSD test (probability level of 5%).** |
