# Supplementary material for: Salt stress memory in tall fescue: Interaction of different stress stages, pollination system and genetic diversity
Source: PLoS One. 2024 Sep 12;19(9):e0310061. doi: 10.1371/journal.pone.0310061 (PMC11392345; doi:10.1371/journal.pone.0310061)
Supplement: S3 Fig — Mean comparison of relative water content for the interaction of two different pollination systems (selfed (S1) and open-pollinated (OP)) and two salinity treatments (C and S1) in the first time (a) and in the second time (b) during two years. Mean comparison total chlorophyll for the interaction of two different pollination systems (selfed (S1) and open-pollinated (OP)) and two salinity treatments (C and S1) in the first time (c) and in the second time (d) during two years. Mean comparison of proline content for the interaction of two different pollination systems (selfed (S1) and open-pollinated (OP)) and two salinity treatments (C and S1) in the first time (e) and in the second time (f) during two years. Mean followed by the same letter is not significantly different according to LSD test (probability level of 5%). (DOCX) [file pone.0310061.s003.docx]

|  |  |
| --- | --- |
|  |  |
|  |  |
| **S3 Fig. Mean comparison of relative water content for the interaction of two different pollination systems (selfed (S_1_) and open-pollinated (OP)) and two salinity treatments (C and S_1_) in the first time (a) and in the second time (b) during two years. Mean comparison total chlorophyll for the interaction of two different pollination systems (selfed (S_1_) and open-pollinated (OP)) and two salinity treatments (C and S_1_) in the first time (c) and in the second time (d) during two years. Mean comparison of proline content for the interaction of two different pollination systems (selfed (S_1_) and open-pollinated (OP)) and two salinity treatments (C and S_1_) in the first time (e) and in the second time (f) during two years. Mean followed by the same letter is not significantly different according to LSD test (probability level of 5%).** | |
