# Supplementary material for: Salt stress memory in tall fescue: Interaction of different stress stages, pollination system and genetic diversity
Source: PLoS One. 2024 Sep 12;19(9):e0310061. doi: 10.1371/journal.pone.0310061 (PMC11392345; doi:10.1371/journal.pone.0310061)
Supplement: S1 Table — (DOCX) [file pone.0310061.s004.docx]

| **S1Table. The formula of different spectral reflectance indices, which were used in this study.** | |
| --- | --- |
| **Spectral reflectance indices** | **Formula†** |
| Normalized difference vegetation index (NDVI) | (R_900_ - R_680_)/ (R_900_ + R_680_) |
| Simple ratio (SR) | R_900_/ R_680_ |
| Water index (WI) | R_970_/ R_900_ |
| Normalized water index (NWI) | (R_970_ - R_900_)/ (R_970_ + R_900_) |
| Ratio analysis of reflectance spectra (RARSa) | R_675_/ (R_650_ * R_700_) |
| Ratio analysis of reflectance spectra (RARSb) | R_760_/ R_500_ |
| Pigment specific simple ratio (PSSR) | R_800_/ R_470_ |
| Pigment specific normalized different (PSND) | (R_800_ - R_470_)/ (R_800_ + R_470_) |
| Structure intensive pigment index (SIPI) | (R_800_ - R_445_)/ (R_800_ + R_445_) |
| Red normalized difference vegetation index (RNDVI) | (R_780_ - R_670_)/ (R_780_ + R_670_) |
| Green normalized difference vegetation index (GNDVI) | (R_780_ - R_550_)/ (R_780_ + R_550_) |
| Photochemical reflectance index (PRI) | (R_531_ - R_570_)/ (R_531_ + R_570_) |
| Normalized difference red edge index (NDRE) | (R_790_ - R_720_)/ (R_790_ + R_720_) |
| Plant senescence reflectance index (PSRI) | (R_680_ - R_500_)/ R_750_ |
| Cartenoid reflectance index (CRI) | ((1/R_510_) - (1/R_700_)) |
| Anthocyanin reflectance index (ARI) | ((1/R_550_) - (1/R_700_)) |
| Green difference vegetation index (GDVI) | NIR-GREEN = (R_900_ - R_510_) |
| RGR | Red/ Green ratio |
| † R and the subindex indicate the reflectance of light at that specific wavelength (in nm). | |
