# Supplementary material for: Salt stress memory in tall fescue: Interaction of different stress stages, pollination system and genetic diversity
Source: PLoS One. 2024 Sep 12;19(9):e0310061. doi: 10.1371/journal.pone.0310061 (PMC11392345; doi:10.1371/journal.pone.0310061)
Supplement: S2 Table — (DOCX) [file pone.0310061.s005.docx]

| **S2Table. Mean squares of morphological traits and relative water content in four tall fescue genotypes and two different pollination systems (selfed (S_1_) and open-pollinated (OP)) in five salinity treatments (C, S_1t1_S_2_, S_1t2_S_2_, S_2_ and H_2_S_2_) evaluated during two years.** | | | | | | | | | |
| --- | --- | --- | --- | --- | --- | --- | --- | --- | --- |
| **Source of variation** | **df** | **CRD** | **PH** | **RWC** | **WFY** | **DFY** | **PHR** | **WFYR** | **DFYR** |
| Year | 1 | 466.8^*^ | 11850.8^**^ | 2315.6 ^ns^ | 0.251^**^ | 0.035^*^ | 12798.5^**^ | 1.89^**^ | 0.575^**^ |
| Rep (year) | 2 | 6.95 | 48.70 | 949.6 | 0.000 | 0.000 | 84.25 | 0.000 | 0.000 |
| Genotype (G) | 3 | 23.71^**^ | 56.75^**^ | 337.92^**^ | 0.011^**^ | 0.001^**^ | 61.70^**^ | 0.059^**^ | 0.021^**^ |
| Pollination (P) | 1 | 15.06^**^ | 45.15^**^ | 734.92^**^ | 0.070^**^ | 0.019^**^ | 178.50^**^ | 1.09^**^ | 0.516^**^ |
| Treatment (T) | 4 | 4.51^*^ | 37.69^**^ | 224.85^*^ | 0.002^**^ | 0.003^**^ | 47.93^**^ | 0.052^**^ | 0.017^**^ |
| G⨯P | 3 | 16.50^**^ | 23.85^**^ | 195.41^*^ | 0.006^**^ | 0.002^**^ | 30.46^*^ | 0.061^**^ | 0.017^**^ |
| G⨯T | 12 | 3.21 ^ns^ | 13.81^**^ | 930.89^**^ | 0.007^**^ | 0.004^**^ | 25.91^**^ | 0.043^**^ | 0.015^**^ |
| P⨯T | 4 | 11.95^**^ | 71.71^**^ | 589.90^**^ | 0.022^**^ | 0.004^**^ | 90.28^**^ | 0.018^**^ | 0.006^**^ |
| G⨯P⨯T | 12 | 4.98^**^ | 26.75^**^ | 468.79^**^ | 0.013^**^ | 0.005^**^ | 38.02^**^ | 0.045^**^ | 0.011^**^ |
| Y⨯G | 3 | 6.06^*^ | 90.37^**^ | 146.15 ^ns^ | 0.024^**^ | 0.005^**^ | 123.13^**^ | 0.042^**^ | 0.015^**^ |
| Y⨯P | 1 | 3.27 ^ns^ | 58.80^**^ | 243.32 ^ns^ | 0.036^**^ | 0.023^**^ | 54.05^*^ | 0.43^**^ | 0.185^**^ |
| Y⨯T | 4 | 6.84^**^ | 92.99^**^ | 348.61^**^ | 0.016^**^ | 0.008^**^ | 10.56 ^ns^ | 0.053^**^ | 0.010^**^ |
| Y⨯G⨯P | 3 | 11.89^**^ | 68.17^**^ | 80.70 ^ns^ | 0.019^**^ | 0.010^**^ | 12.73 ^ns^ | 0.056^**^ | 0.026^**^ |
| Y⨯G⨯T | 12 | 7.72^**^ | 31.56^**^ | 156.72^*^ | 0.006^**^ | 0.002^**^ | 24.23^**^ | 0.035^**^ | 0.011^**^ |
| Y⨯P⨯T | 4 | 14.06^**^ | 20.99^**^ | 46.16 ^ns^ | 0.031^**^ | 0.010^**^ | 69.46^**^ | 0.032^**^ | 0.011^**^ |
| Y⨯G⨯P⨯T | 12 | 3.92^*^ | 35.73^**^ | 89.43 ^ns^ | 0.011^**^ | 0.003^**^ | 43.37^**^ | 0.038^**^ | 0.009^**^ |
| Error | 78 | 1.71 | 4.87 | 68.88 | 0.000 | 0.000 | 8.00 | 0.001 | 0.000 |
| Coefficient of variation (%) |  | 29.00 | 17.49 | 15.49 | 12.98 | 17.13 | 25.91 | 17.67 | 11.34 |
| * and ** show significance at the 0.05 and 0.01 probability levels, respectively. ns: not significant.  CRD, crown diameter; PH, plant height; RWC, relative water content; WFY, wet forage yield; DFY, dry forage yield; PHR, plant height in recovery; WFYR, wet forage yield in recovery; DFYR, dry forage yield in recovery. | | | | | | | | | |
