# Supplementary material for: Salt stress memory in tall fescue: Interaction of different stress stages, pollination system and genetic diversity
Source: PLoS One. 2024 Sep 12;19(9):e0310061. doi: 10.1371/journal.pone.0310061 (PMC11392345; doi:10.1371/journal.pone.0310061)
Supplement: S3 Table — (DOCX) [file pone.0310061.s006.docx]

| **S3Table. Mean squares of physiological traits in four tall fescue genotypes and two different pollination systems (selfed (S_1_) and open-pollinated (OP)) in five salinity treatments (C, S_1t1_S_2_, S_1t2_S_2_, S_2_ and H_2_S_2_) evaluated two years.** | | | | | | | | | | | |
| --- | --- | --- | --- | --- | --- | --- | --- | --- | --- | --- | --- |
| **Source of variation** | **df** | **Chla** | **Chlb** | **Car** | **Tchl** | **Chl a/b** | **Tchl/Car** | **Pro** | **CAT** | **APX** | **POX** |
| Year | 1 | 0.77^*^ | 0.037 ^ns^ | 0.147^*^ | 1.15^*^ | 2.92^*^ | 1.30 ^ns^ | 0.28 ^ns^ | 0.392^*^ | 0.007^*^ | 0.000 ^ns^ |
| Rep (year) | 2 | 0.017 | 0.002 | 0.004 | 0.033 | 0.071 | 0.170 | 2.62 | 0.017 | 0.000 | 0.008 |
| Genotype (G) | 3 | 0.000 ^ns^ | 0.007^**^ | 0.001 ^ns^ | 0.009 ^ns^ | 0.60^**^ | 0.667^**^ | 2.90^**^ | 0.052^**^ | 0.006^**^ | 0.209^**^ |
| Pollination (P) | 1 | 0.205^**^ | 0.033^**^ | 0.030^**^ | 0.405^**^ | 0.73^**^ | 0.606 ^ns^ | 4.54^**^ | 0.002 ^ns^ | 0.001^**^ | 0.274^**^ |
| Treatment (T) | 4 | 0.699^**^ | 0.075^**^ | 0.026^**^ | 1.19^**^ | 2.73^**^ | 11.99^**^ | 0.431^**^ | 0.214^**^ | 0.001^**^ | 0.143^**^ |
| G⨯P | 3 | 0.056^**^ | 0.017^**^ | 0.009^**^ | 0.105^**^ | 1.71^**^ | 0.074 ^ns^ | 0.471^**^ | 0.086^**^ | 0.001^**^ | 0.204^**^ |
| G⨯T | 12 | 0.044^**^ | 0.005^**^ | 0.004^**^ | 0.069^**^ | 0.82^**^ | 0.392^*^ | 0.72^**^ | 0.135^**^ | 0.002^**^ | 0.111^**^ |
| P⨯T | 4 | 0.097^**^ | 0.010^**^ | 0.007^**^ | 0.154^**^ | 0.82^**^ | 0.332 ^ns^ | 0.86^**^ | 0.273^**^ | 0.003^**^ | 0.086^**^ |
| G⨯P⨯T | 12 | 0.042^**^ | 0.009^**^ | 0.005^**^ | 0.077^**^ | 0.62^**^ | 0.412^**^ | 0.78^**^ | 0.068^**^ | 0.002^**^ | 0.057^**^ |
| Y⨯G | 3 | 0.009 ^ns^ | 0.000 ^ns^ | 0.003^**^ | 0.009 ^ns^ | 0.34^**^ | 1.53^**^ | 0.65^*^ | 0.109^**^ | 0.000^**^ | 0.026^**^ |
| Y⨯P | 1 | 0.000 ^ns^ | 0.016^**^ | 0.001 ^ns^ | 0.014 ^ns^ | 1.86^**^ | 0.158 ^ns^ | 0.61 ^ns^ | 0.015 ^ns^ | 0.001^**^ | 0.003 ^ns^ |
| Y⨯T | 4 | 0.158^**^ | 0.042^**^ | 0.023^**^ | 0.360^**^ | 0.27^**^ | 3.67^**^ | 1.02^**^ | 0.107^**^ | 0.000^**^ | 0.019^**^ |
| Y⨯G⨯P | 3 | 0.027^*^ | 0.012^**^ | 0.001 ^ns^ | 0.059^**^ | 0.87^**^ | 0.301 ^ns^ | 0.35 ^ns^ | 0.021^**^ | 0.001^**^ | 0.009^**^ |
| Y⨯G⨯T | 12 | 0.031^**^ | 0.005^**^ | 0.004^**^ | 0.054^**^ | 0.55^**^ | 0.650^**^ | 0.55^**^ | 0.065^**^ | 0.001^**^ | 0.042^**^ |
| Y⨯P⨯T | 4 | 0.067^**^ | 0.002^*^ | 0.007^**^ | 0.080^**^ | 1.01^**^ | 0.405 ^ns^ | 0.34 ^ns^ | 0.004 ^ns^ | 0.001^**^ | 0.006^*^ |
| Y⨯G⨯P⨯T | 12 | 0.036^**^ | 0.003^**^ | 0.006^**^ | 0.053^**^ | 0.35^**^ | 0.608^**^ | 0.50^**^ | 0.031^**^ | 0.001^**^ | 0.034^**^ |
| Error | 78 | 0.006 | 0.000 | 0.001 | 0.012 | 0.045 | 0.165 | 0.170 | 0.004 | 0.000 | 0.001 |
| Coefficient of variation (%) |  | 16.56 | 14.79 | 16.77 | 15.74 | 8.85 | 12.33 | 23.12 | 24.95 | 22.38 | 24.20 |
| * and ** show significance at the 0.05 and 0.01 probability levels, respectively. ns: not significant.  Chla, chlorophyll a content; Chlb, chlorophyll b content; Car, carotenoid content; Tchl, total chlorophyll; Chl a/b, ratio of Chla/Chlb; Tchl/Car, ratio of Tchl/Car; Pro, proline content; CAT, catalase activity; APX, ascorbate peroxidase activity; POX, peroxidase activity. | | | | | | | | | | | |
