# Supplementary material for: Salt stress memory in tall fescue: Interaction of different stress stages, pollination system and genetic diversity
Source: PLoS One. 2024 Sep 12;19(9):e0310061. doi: 10.1371/journal.pone.0310061 (PMC11392345; doi:10.1371/journal.pone.0310061)
Supplement: S4 Table — (DOCX) [file pone.0310061.s007.docx]

| **S4Table. Mean squares of root traits in four tall fescue genotypes and two different pollination systems (selfed (S_1_) and open-pollinated (OP)) in five salinity treatments (C, S_1t1_S_2_, S_1t2_S_2_, S_2_ and H_2_S_2_) evaluated during two years.** | | | | | | | | | | |
| --- | --- | --- | --- | --- | --- | --- | --- | --- | --- | --- |
| **Source of variation** | **df** | **RL** | **RV(A)** | **RV(G)** | **RA** | **RCL** | **RWW** | **RDW** | **R/S** | **R/SR** |
| Year | 1 | 1086.8 ^ns^ | 6.61^*^ | 4.10^**^ | 3530.2^**^ | 2978004^**^ | 5.27^*^ | 0.192^*^ | 146.0^**^ | 77.63^**^ |
| Rep (year) | 2 | 198.7 | 0.101 | 0.004 | 80.31 | 13827.0 | 0.086 | 0.006 | 0.08 | 0.014 |
| Genotype (G) | 3 | 591.3^**^ | 0.722^**^ | 0.67^**^ | 6228.9^**^ | 512488.4^**^ | 0.507^**^ | 0.022^**^ | 11.57^**^ | 14.92^**^ |
| Pollination (P) | 1 | 693.05^**^ | 2.66^**^ | 4.50^**^ | 35561.3^**^ | 2825983^**^ | 2.44^**^ | 0.311^**^ | 13.35^**^ | 0.225 ^ns^ |
| Treatment (T) | 4 | 190.05^**^ | 0.131^**^ | 0.050^**^ | 460.8^**^ | 30130.7^**^ | 0.164^**^ | 0.015^**^ | 5.55^**^ | 21.31^**^ |
| G⨯P | 3 | 697.75^**^ | 0.754^**^ | 0.806^**^ | 6356.0^**^ | 527177^**^ | 0.629^**^ | 0.017^**^ | 7.40^**^ | 6.76^**^ |
| G⨯T | 12 | 154.21^**^ | 0.104^**^ | 0.190^**^ | 1485.9^**^ | 118985^**^ | 0.114^**^ | 0.006^**^ | 17.57^**^ | 10.61^**^ |
| P⨯T | 4 | 185.68^**^ | 0.141^**^ | 0.291^**^ | 2398.6^**^ | 189045^**^ | 0.310^**^ | 0.012^**^ | 11.88^**^ | 7.25^**^ |
| G⨯P⨯T | 12 | 167.15^**^ | 0.070^**^ | 0.097^**^ | 888.2^**^ | 74201.5^**^ | 0.071^**^ | 0.005^**^ | 21.95^**^ | 19.60^**^ |
| Y⨯G | 3 | 174.00^**^ | 0.548^**^ | 0.524^**^ | 4345.2^**^ | 343710.4^**^ | 0.397^**^ | 0.006^**^ | 16.23^**^ | 12.56^**^ |
| Y⨯P | 1 | 438.90^**^ | 1.68^**^ | 1.11^**^ | 9304.6^**^ | 766260.3^**^ | 0.428^**^ | 0.031^**^ | 1.35^**^ | 1.57^**^ |
| Y⨯T | 4 | 115.80^**^ | 0.075^**^ | 0.078^**^ | 647.1^**^ | 52906.5^**^ | 0.197^**^ | 0.006^**^ | 8.61^**^ | 24.30^**^ |
| Y⨯G⨯P | 3 | 310.07^**^ | 0.659^**^ | 0.684^**^ | 5828.7^**^ | 476992.9^**^ | 0.607^**^ | 0.007^**^ | 9.84^**^ | 11.58^**^ |
| Y⨯G⨯T | 12 | 103.52^**^ | 0.072^**^ | 0.159^**^ | 1280.6^**^ | 98749.6^**^ | 0.115^**^ | 0.004^**^ | 15.75^**^ | 10.99^**^ |
| Y⨯P⨯T | 4 | 163.90^**^ | 0.052^**^ | 0.259^**^ | 2169.4^**^ | 176723.4^**^ | 0.291^**^ | 0.014^**^ | 14.65^**^ | 5.43^**^ |
| Y⨯G⨯P⨯T | 12 | 253.96^**^ | 0.058^**^ | 0.211^**^ | 1753.8^**^ | 140486.8^**^ | 0.100^**^ | 0.002^**^ | 19.26^**^ | 18.77^**^ |
| Error | 78 | 25.21 | 0.007 | 0.006 | 47.58 | 3750.7 | 0.009 | 0.000 | 0.135 | 0.112 |
| Coefficient of variation (%) |  | 22.23 | 28.87 | 22.99 | 21.88 | 21.49 | 29.70 | 27.41 | 17.32 | 27.28 |
| * and ** show significance at the 0.05 and 0.01 probability levels, respectively. ns: not significant.  RL, root length; RV(A), root volume (Archimedes); RV(G), root volume (Giaroot); RA, root area; RCL, root cumulative length; RWW, root wet weight; RDW, root dry weight; R/S, root to shoot ratio; R/SR, root to shoot ratio in recovery. | | | | | | | | | | |
