# Supplementary material for: Salt stress memory in tall fescue: Interaction of different stress stages, pollination system and genetic diversity
Source: PLoS One. 2024 Sep 12;19(9):e0310061. doi: 10.1371/journal.pone.0310061 (PMC11392345; doi:10.1371/journal.pone.0310061)
Supplement: S5 Table — (DOCX) [file pone.0310061.s008.docx]

| **S5Table. Correlation coefficients among spectral reflectance indices based on the average of two years for eight genotypes (1MOP, 1MS_1_, 3MOP, 3MPS_1_, 11MOP, 11MS_1_, 21MOP and 21MS_1_), five salinity treatments (C, S_1t1_S_2_, S_1t2_S_2_, S_2_ and H_2_S_2_) and two replications (n=80).** | | | | | | | | | | | | | | | | | | |
| --- | --- | --- | --- | --- | --- | --- | --- | --- | --- | --- | --- | --- | --- | --- | --- | --- | --- | --- |
|  | **NDVI** | **SR** | **WI** | **NWI** | **RARSa** | **RARSb** | **PSSR** | **PSND** | **SIPI** | **RNDVI** | **GNDVI** | **PRI** | **NDRE** | **PSRI** | **CRI** | **ARI** | **GDVI** | **RGR** |
| **NDVI** | 1.00 |  |  |  |  |  |  |  |  |  |  |  |  |  |  |  |  |  |
| **SR** | -0.22* | 1.00 |  |  |  |  |  |  |  |  |  |  |  |  |  |  |  |  |
| **WI** | -0.76** | -0.05 | 1.00 |  |  |  |  |  |  |  |  |  |  |  |  |  |  |  |
| **NWI** | -0.78** | 0.04 | 0.98** | 1.00 |  |  |  |  |  |  |  |  |  |  |  |  |  |  |
| **RARSa** | 0.74** | -0.38** | -0.48** | -0.52** | 1.00 |  |  |  |  |  |  |  |  |  |  |  |  |  |
| **RARSb** | 0.51** | 0.13 | -0.41** | -0.40** | 0.24* | 1.00 |  |  |  |  |  |  |  |  |  |  |  |  |
| **PSSR** | 0.65** | -0.06 | -0.44** | -0.45** | 0.27* | 0.47** | 1.00 |  |  |  |  |  |  |  |  |  |  |  |
| **PSND** | 0.87** | -0.21 | -0.59** | -0.62** | 0.71** | 0.57** | 0.62** | 1.00 |  |  |  |  |  |  |  |  |  |  |
| **SIPI** | 0.85** | -0.18 | -0.58** | -0.61** | 0.68** | 0.54** | 0.60** | 0.99** | 1.00 |  |  |  |  |  |  |  |  |  |
| **RNDVI** | 1.00** | -0.20 | -0.77** | -0.78** | 0.73** | 0.52** | 0.64** | 0.88** | 0.84** | 1.00 |  |  |  |  |  |  |  |  |
| **GNDVI** | 0.75** | -0.19 | -0.50** | -0.51** | 0.69** | 0.58** | 0.49** | 0.89** | 0.84** | 0.76** | 1.00 |  |  |  |  |  |  |  |
| **PRI** | 0.67** | -0.29* | -0.47** | -0.50** | 0.91** | 0.21 | 0.16 | 0.68** | 0.66** | 0.66** | 0.68** | 1.00 |  |  |  |  |  |  |
| **NDRE** | 0.69** | -0.11 | -0.50** | -0.52** | 0.74** | 0.54** | 0.35** | 0.76** | 0.72** | 0.70** | 0.90** | 0.72** | 1.00 |  |  |  |  |  |
| **PSRI** | 0.45** | -0.12 | -0.17 | -0.20 | 0.46** | 0.45** | 0.29* | 0.74** | 0.73** | 0.45** | 0.72** | 0.47** | 0.55** | 1.00 |  |  |  |  |
| **CRI** | 0.24* | 0.14 | -0.28* | -0.27* | 0.06 | 0.26* | 0.13 | 0.26* | 0.23* | 0.24* | 0.29* | 0.04 | 0.26* | 0.25* | 1.00 |  |  |  |
| **ARI** | -0.70** | 0.20 | 0.50** | 0.51** | -0.59** | -0.44** | -0.46** | -0.56** | -0.54** | -0.70** | -0.50** | -0.50** | -0.54** | -0.22** | -0.04 | 1.00 |  |  |
| **GDVI** | -0.21 | 0.33** | 0.14 | 0.15 | -0.23* | 0.09 | 0.07 | 0.09 | 0.14 | -0.20 | 0.16 | -0.18 | 0.06 | 0.45** | 0.15 | 0.27* | 1.00 |  |
| **RGR** | 0.44** | -0.75** | -0.02 | -0.10 | 0.55** | 0.16 | 0.37** | 0.43** | 0.38** | 0.43** | 0.44** | 0.39** | 0.35** | 0.35** | -0.10 | -0.48** | -0.19 | 1.00 |
| * and ** show significance at the 0.05 and 0.01 probability levels, respectively.  NDVI, normalized difference vegetation index; SR, simple ratio; WI, water index; NWI, normalized water index; RARSa, ratio analysis of reflectance spectra; RARSb, ratio analysis of reflectance spectra; PSSR, pigment specific simple ratio; PSND, pigment specific normalized different; SIPI, structure intensive pigment index; RNDVI, red normalized difference vegetation index; GNDVI, green normalized difference vegetation index; PRI, photochemical reflectance index; NDRE, normalized difference red edge index; PSRI, plant senescence reflectance index; CRI, cartenoid reflectance index; ARI, anthocyanin reflectance index; GDVI, green difference vegetation index; RGR, red/ green ratio. | | | | | | | | | | | | | | | | | | |
