# Supplementary material for: Salt stress memory in tall fescue: Interaction of different stress stages, pollination system and genetic diversity
Source: PLoS One. 2024 Sep 12;19(9):e0310061. doi: 10.1371/journal.pone.0310061 (PMC11392345; doi:10.1371/journal.pone.0310061)
Supplement: S6 Table — (DOCX) [file pone.0310061.s009.docx]

| **S6Table. The first two principal components (PC) loadings for the measured traits on 8 tall fescue genotypes in five treatment levels (C, S_1t1_S_2_, S_1t2_S_2_, S_2_ and H_2_S_2_) during two years.** | | | | | | | | | | | | | | | |
| --- | --- | --- | --- | --- | --- | --- | --- | --- | --- | --- | --- | --- | --- | --- | --- |
| **Characters** |  | **C** | |  | **S_1t1_S_2_** | |  | **S_1t2_S_2_** | |  | **S_2_** | |  | **H_2_S_2_** | |
|  |  | **PC1** | **PC2** |  | **PC1** | **PC2** |  | **PC1** | **PC2** |  | **PC1** | **PC2** |  | **PC1** | **PC2** |
| CRD (cm) |  | 0.298 | -0.165 |  | -0.183 | -0.073 |  | 0.205 | 0.079 |  | -0.009 | -0.356 |  | 0.005 | 0.016 |
| PH (cm) |  | -0.152 | -0.164 |  | 0.143 | 0.330 |  | 0.069 | 0.160 |  | 0.179 | 0.271 |  | 0.137 | -0.030 |
| DFY (g/plant) |  | 0.031 | 0.040 |  | -0.157 | 0.193 |  | 0.282 | 0.077 |  | 0.297 | 0.123 |  | -0.224 | -0.240 |
| PHR (cm) |  | -0.059 | -0.103 |  | 0.040 | 0.418 |  | -0.103 | 0.179 |  | 0.087 | -0.087 |  | 0.224 | 0.183 |
| DFYR (g/plant) |  | 0.338 | 0.057 |  | -0.283 | 0.119 |  | 0.269 | 0.174 |  | 0.338 | 0.101 |  | -0.336 | -0.057 |
| RWC (%) |  | 0.054 | 0.006 |  | -0.261 | 0.148 |  | -0.027 | -0.299 |  | -0.190 | 0.093 |  | -0.149 | -0.208 |
| Chla (mg/g leaf) |  | -0.133 | -0.372 |  | -0.196 | 0.036 |  | -0.081 | 0.383 |  | -0.011 | 0.327 |  | -0.115 | 0.352 |
| Chlb (mg/g leaf) |  | -0.069 | -0.398 |  | -0.239 | -0.102 |  | 0.246 | 0.163 |  | -0.056 | 0.256 |  | -0.076 | 0.353 |
| Car (mg/g leaf) |  | -0.108 | -0.325 |  | -0.209 | -0.034 |  | -0.009 | 0.313 |  | -0.034 | 0.351 |  | -0.089 | 0.384 |
| Tchl (mg/g leaf) |  | -0.114 | -0.383 |  | -0.218 | -0.001 |  | -0.019 | 0.385 |  | -0.024 | 0.319 |  | -0.108 | 0.383 |
| Chl a/b |  | -0.127 | 0.290 |  | 0.075 | 0.168 |  | -0.194 | 0.326 |  | 0.105 | 0.035 |  | -0.073 | -0.152 |
| Tchl/Car |  | 0.032 | -0.351 |  | -0.191 | 0.185 |  | 0.113 | 0.313 |  | 0.042 | -0.078 |  | -0.156 | 0.294 |
| Pro (µmol/g leaf) |  | -0.092 | 0.189 |  | -0.204 | 0.312 |  | 0.027 | 0.110 |  | 0.069 | 0.162 |  | -0.065 | -0.285 |
| CAT (µmol min^-1^ mg^-1^ protein) |  | -0.079 | 0.086 |  | -0.112 | -0.359 |  | 0.268 | -0.032 |  | -0.183 | 0.243 |  | 0.194 | -0.122 |
| APX (µmol min^-1^ mg^-1^ protein) |  | 0.025 | 0.196 |  | 0.068 | -0.167 |  | 0.221 | -0.019 |  | -0.113 | 0.169 |  | -0.081 | -0.208 |
| POX (µmol min^-1^ mg^-1^ protein) |  | 0.034 | 0.069 |  | -0.177 | -0.307 |  | 0.284 | 0.044 |  | -0.251 | 0.167 |  | 0.129 | 0.216 |
| RL (cm) |  | 0.171 | -0.242 |  | -0.227 | -0.100 |  | -0.074 | -0.140 |  | 0.189 | -0.289 |  | -0.061 | -0.068 |
| RV(A) (cm^3^/plant) |  | 0.335 | 0.026 |  | -0.268 | -0.087 |  | 0.305 | -0.080 |  | 0.341 | -0.024 |  | -0.323 | 0.000 |
| RV(G) (cm^3^/plant) |  | 0.361 | -0.055 |  | -0.280 | 0.068 |  | 0.300 | -0.076 |  | 0.330 | 0.042 |  | -0.330 | 0.022 |
| RA (cm^2^/plant) |  | 0.361 | -0.057 |  | -0.274 | 0.070 |  | 0.300 | -0.085 |  | 0.332 | 0.040 |  | -0.330 | 0.018 |
| RCL (cm/plant) |  | 0.361 | -0.060 |  | -0.273 | 0.065 |  | 0.299 | -0.093 |  | 0.334 | 0.036 |  | -0.330 | 0.013 |
| RDW (g/plant) |  | 0.320 | 0.005 |  | -0.299 | -0.027 |  | 0.298 | -0.004 |  | 0.326 | 0.148 |  | -0.311 | -0.036 |
| R/S |  | 0.213 | -0.031 |  | -0.063 | -0.326 |  | 0.082 | 0.269 |  | -0.014 | -0.129 |  | -0.275 | -0.010 |
| R/SR |  | -0.050 | -0.116 |  | 0.055 | -0.268 |  | 0.053 | 0.217 |  | -0.026 | -0.286 |  | -0.030 | 0.112 |
| Eigenvalue |  | 7.27 | 5.39 |  | 10.29 | 4.18 |  | 10.02 | 5.46 |  | 7.49 | 5.30 |  | 8.40 | 5.73 |
| Percent of variation |  | 30.29 | 22.47 |  | 42.89 | 21.60 |  | 41.75 | 22.78 |  | 31.22 | 22.11 |  | 35.01 | 23.91 |
| Cumulative percentage |  | 30.29 | 52.77 |  | 42.89 | 64.49 |  | 41.75 | 64.53 |  | 31.22 | 53.34 |  | 35.01 | 58.92 |
| CRD, crown diameter; PH, plant height; DFY, dry forage yield; PHR, plant height in recovery; DFYR, dry forage yield in recovery; RWC, relative water content; Chla, chlorophyll a content; Chlb, chlorophyll b content; Car, carotenoid content; Tchl, total chlorophyll; Chla/b, ratio of Chla/Chlb; Tchl/Car, ratio of Tchl/Car; Pro, proline content; CAT, catalase activity; APX, ascorbate peroxidase activity; POX, peroxidase activity; RL, root length; RV(A), root volume (Archimedes); RV(G), root volume (Giaroot); RA, root area; RCL, root cumulative length; RDW, root dry weight; R/S, root to shoot ratio; R/SR, root to shoot ratio in recovery. | | | | | | | | | | | | | | | |
